# Rehabilitation of back pain in the pediatric population: a mixed studies systematic review

**DOI:** 10.1186/s12998-024-00538-z

**Published:** 2024-05-08

**Authors:** Hainan Yu, Danielle Southerst, Jessica J. Wong, Leslie Verville, Gaelan Connell, Lauren Ead, Silvano Mior, Lise Hestbaek, Michael Swain, Ginny Brunton, Heather M. Shearer, Efrosini Papaconstantinou, Daphne To, Darrin Germann, Katie Pohlman, Christine Cedraschi, Carol Cancelliere

**Affiliations:** 1 Institute for Disability and Rehabilitation Research and Faculty of Health Sciences, Ontario Tech University, Oshawa, ON L1G 0C5 Canada; 2 Department of Research and Innovation, Canadian Memorial Chiropractic College, Toronto, Canada; 3 The Chiropractic Knowledge Hub, Odense, Denmark; 4 Department of Sports Science and Clinical Biomechanics, University of Southern Denmark, Odense, Denmark; 5 Department of Chiropractic, Faculty of Medicine, Health and Human Sciences, Macquarie University, Macquarie Park, Australia; 6 Research Center, Parker University, Dallas, TX USA; 7 Division of General Medical Rehabilitation, University of Geneva, Geneva, Switzerland; 8 Division of Clinical Pharmacology & Toxicology, Multidisciplinary Pain Centre, Geneva University Hospitals, Geneva, Switzerland

**Keywords:** Children, Adolescents, Low back pain, Thoracic pain, Spinal manipulation, Exercise, Rehabilitation

## Abstract

**Background:**

A significant proportion of children and adolescents experience back pain. However, a comprehensive systematic review on the effectiveness of rehabilitation interventions is lacking.

**Objectives:**

To evaluate benefits and harms of rehabilitation interventions for non-specific low back pain (LBP) or thoracic spine pain in the pediatric population.

**Methods:**

Seven bibliographic electronic databases were searched from inception to June 16, 2023. Moreover, reference lists of relevant studies and systematic reviews, three targeted websites, and the WHO International Clinical Trials Registry Platform were searched. Paired reviewers independently conducted screening, assessed risk of bias, and extracted data related to study characteristics, methodology, subjects, and results. Certainty of evidence was evaluated based on the GRADE approach.

**Results:**

We screened 8461 citations and 307 full-text articles. Ten quantitative studies (i.e., 8 RCTs, 2 non-randomized clinical trials) and one qualitative study were included. With very low to moderate certainty evidence, in adolescents with LBP, spinal manipulation (1–2 sessions/week over 12 weeks, 1 RCT) plus exercise may be associated with a greater likelihood of experiencing clinically important pain reduction versus exercise alone; and group-based exercise over 8 weeks (2 RCTs and 1 non-randomized trial) may reduce pain intensity. The qualitative study found information provided via education/advice and compliance of treatment were related to effective treatment. No economic studies or studies examining thoracic spine pain were identified.

**Conclusions:**

Spinal manipulation and group-based exercise may be beneficial in reducing LBP intensity in adolescents. Education should be provided as part of a care program. The overall evidence is sparse. Methodologically rigorous studies are needed.

**Trial registration:**

CRD42019135009 (PROSPERO)

**Supplementary Information:**

The online version contains supplementary material available at 10.1186/s12998-024-00538-z.

## Introduction

A significant proportion of children and adolescents experience back pain (i.e., thoracic spine pain and low back pain). A systematic review reported that the annual prevalence of low back pain (LBP) is 33.6% (95%CI 26.9%, 41%) in children and adolescents (≤ 18 years old) [1]. The lifetime prevalence of thoracic spine pain (TSP) varies from 9.5% to 72% in children and adolescents [2]. Most episodes of spinal pain (including neck and back pain) are brief in children and adolescents; however, 31% have a recurrence of spinal pain over one year and up to 25% have three or more episodes over one year, and approximately 13% reported episodes lasting five or more weeks [3, 4]. In a cross-sectional international study (650,851 participants), the prevalence of back pain in adolescents increases from early to late adolescents, and into young adulthood [5].

Two recent systematic reviews assessed the effectiveness of manual therapy to treat a number of conditions including back pain in children and adolescents, but a judgement of effectiveness was precluded due to limited and low-quality evidence (e.g., 4 studies including one case series and one cohort study without a control group in Prevost et al. [2019] review and only one study in Driehuis et al. [2019] review) [6, 7]. Another systematic review and meta-analysis evaluating the effectiveness of conservative interventions for LBP in children and adolescents reported that exercise interventions may be promising for improving pain intensity in children compared to no treatment. However, this review included studies with mixed neck, shoulder and back pain participants, and given their literature search is outdated (included studies until 2013), this evidence needs updating [8].

To inform healthcare professionals in a variety of clinical, rehabilitation or community settings for evidence-based care, we conducted an integrative systematic review of quantitative, qualitative, and economic evidence regarding the rehabilitative management of back pain (including TSP and LBP) in children and adolescents aged 19 years and younger.

## Methods

We registered our protocol on the International Prospective Register of Systematic Reviews (PROSPERO) (CRD42019135009) and published it in BMJ Open [9]. We reported our systematic review according to the Preferred Reporting Items for Systematic Reviews and Meta-Analyses (PRISMA) statement (Additional file 1) [10], and the Synthesis Without Meta-analysis (SWiM) reporting guideline [10].

### Eligibility criteria

Our review included studies that: 1) enrolled children and adolescents (aged 19 years or younger) with non-specific LBP or TSP; 2) investigated rehabilitation interventions (Table 1); 3) compared the intervention of interest with other conservative interventions, placebo or sham, wait list, standard care, and no intervention (including intervention of interest as an addition to active comparison interventions where the attributable effect of the comparison interventions can be isolated); and 4) reported patient-important outcomes related to functioning as described by the International Classification of Functioning, Disability and Health (ICF) framework [11] domains *body functions and structures* (to describe a child’s impairment such as pain), and *activities and participation* (to describe a child’s functional status and involvement in life situations), adverse events, cost measures or qualitative outcomes (Table 2). We used the Convention on the Rights of the Child and the WHO definitions of children (< 18 years of age) and adolescents (10–19 years of age) [12, 13].

**Table 1:** Examples of rehabilitation interventions

**Intervention**	**Definition**	**Examples**
Acupuncture	Any body-needling, moxibustion, electric acupuncture, laser acupuncture, microsystem acupuncture, and acupressure	• Traditional needling • Dry needling • Burning of specific herbs • Electro-acupuncture • Photo-acupuncture
Assistive devices	Any item, piece of equipment or product system, used to increase, maintain, or improve the functional capabilities of people with disabilities	• Walking aids • Orthoses • Braces • Wheelchairs
Exercise	A subcategory of physical activity that is planned, structured, repetitive, and purposeful; can be supervised (e.g., by a healthcare professional) or unsupervised	• Stretching • Strengthening • Range of motion exercises • Aerobic (e.g., swimming, cycling, walking, running) • Anaerobic (e.g., jumping, sprinting, weight lifting)
Manual therapies	- Manipulation: Techniques incorporating a high-velocity low-amplitude impulse or thrust applied at or near the end of a joint’s passive range of motion - Mobilization: Techniques incorporating a low-velocity and small or large amplitude oscillatory movement, within a joint’s passive range of motion - Traction: Manual or mechanically assisted application of an intermittent or continuous distractive force - Soft tissue therapy: A mechanical form of therapy where soft-tissue structures are pressed and kneaded, using physical contact with the hand or mechanical device	• Lumbar manipulation, mobilization, or traction • Massage • Muscle energy technique • Strain-counterstrain
Modifications to environment		• Ergonomic interventions at school or work
Passive physical modalities	A form of cold, heat, or light application affecting the body at the skin level or ultrasonic or electromagnetic radiation affecting structures beneath the skin surface: - Passive assistive devices: Device to encourage immobilization in anatomic positions or actively inhibit or prevent movement	• Heat application: heat pack, hydrotherapy • Cryotherapy: cold pack, vapocoolant spray • Low-level laser • Electrical muscle stimulation • Pulsed electromagnetic therapy
Patient or caregiver education and self-management strategies (structured or unstructured)	Teaching patients skills that they can use to manage their health condition	• Learning disease-specific information • Learning general managing skills (e.g., problem-solving, finding and using community resources, working with healthcare team) • Learning strategies to increase confidence (i.e., self-efficacy) in ability to engage in behaviours that are needed to manage their condition on a daily basis • Adequate peer role models and support networks that facilitate the initiation and maintenance of desired behavioural changes
Pharmacological interventions	A substance used in treating disease or relieving pain	• Acetaminophen • Nonsteroidal anti-inflammatory drugs • Muscle relaxants • Antidepressants
Psychological interventions	Activities used to modify behaviour, emotional state, or feelings	• Cognitive behavioural therapy • Counselling • Social network and environment-based therapies • Psychoeducational interventions • Mindfulness meditation

**Table 2:** Research questions, population, intervention, comparison, outcome and study type

	**Included**	**Excluded**
*Research question 1: What is the effectiveness and safety of rehabilitation interventions for improving pain, functioning, and health outcomes in children and adolescents with back pain?*		
Population	Children and adolescents with low back pain, thoracic spine pain, mechanical back pain, lumbago, lumbar sprain or strain, back sprain or strain, lumbopelvic pain, lumbar radiculopathy, lumbar disc herniation, lumbar spondylolysis, sacroiliac syndrome or sciatica in any duration	(1) Back pain attributed to major structural or systemic pathology (e.g., fracture, infection, tumour, osteoporosis, inflammatory arthritides, cauda equina syndrome, neuromuscular disease, myelopathy and scoliosis) (2) Back pain attributed to a non-spine-related condition that might refer pain to the chest wall (e.g., heart, lung or esophagus conditions)
Intervention	Rehabilitation interventions including pharmacological, non-pharmacological, and psychological interventions delivered by various healthcare providers including, but not limited to, general practitioners, nurses, physiotherapists, chiropractors, occupational therapists, psychologists and registered massage therapists	Surgical interventions, and interventions solely conducted at the societal level, such as barrier removal initiatives (e.g., fitting a ramp to a public building)
Comparison	Other conservative interventions, placebo or sham, wait list, standard care, and no intervention or intervention of interest as an addition to active comparison interventions where the attributable effect of the comparison interventions can be isolated	
Outcome	1. Outcomes related to *body functions and structures* to describe a child’s impairment: e.g., pain intensity, frequency, duration; range of motion; psychological outcomes such as depression and anxiety Examples of outcome measures: NRS, VAS, Faces Pain Scale—Revised;(Hicks et al*.,* 2001, Michaleff et al*.,* 2017) goniometer, Revised Child Anxiety and Depression Scale,(Chorpita et al*.,* 2000) State-Trait Anxiety Inventory for Children,(Spielberger 1973) PROMIS Pediatric Self Report Scale 2. Outcomes related to *activities and participation* to describe a child’s functional status and involvement in life situations: e.g., disability, communication, mobility, interpersonal interactions, preferences, self-care, learning, applying knowledge, return to activities/school Examples of outcome measures: Modified Oswestry Low Back Pain Disability Questionnaire,(Fairbank et al*.,* 1980) KIDSCREEN-52,(Ravens-Sieberer et al*.,* 2008) Pediatric Quality of Life Inventory(Varni et al*.,* 2001) 3. Adverse events: any unfavourable sign, symptom, or disease temporarily associated with the treatment, whether or not caused by the treatment.(Pohlman et al*.,* 2014) We will also consider indirect harms, where the use of an intervention delays a diagnosis or treatment, and such delay holds a potential harm.(Zorzela et al*.,* 2014)	
Study design	Randomized controlled trials Cohort studies Case–control studies Mixed methods studies (quantitative component)	
*Research question 2: What are the patients’, caregivers’ and providers’ experiences, preferences, expectations and valued outcomes regarding rehabilitation interventions for back pain?*		
Outcome	Experiences, preferences, expectations, valued outcomes	
Study design	Qualitative studies (e.g., phenomenology, grounded theory, ethnography, action research, descriptive qualitative studies) Mixed-methods studies (qualitative component)	
*Research question 3: What is the cost-effectiveness of rehabilitation interventions for improving pain, functioning, and health outcomes in children and adolescents with back pain?*		
Outcome	Direct costs: resources consumed or saved by an intervention Indirect costs: productivity gains or losses (e.g., time consumed or freed by the intervention) Economic health outcomes: QALY, ICER, NMB Intangible: e.g., pain or suffering saved or brought on by an intervention	
Study design	Full economic evaluations (trial- and model-based): cost-effectiveness, cost-utility, cost–benefit, cost-consequences	

*ICER* incremental cost-effectiveness ratio, *NMB* measure of net monetary benefit, *NRS* Numerical Rating Scale, *PROMIS* Patient-Reported Outcomes Measurement Information System, *QALY* quality adjusted life years, *VAS* Visual Analogue Scale

The rehabilitation process is designed to assist individuals in regaining, improving, or maximizing functioning and quality of life after experiencing injuries, surgeries, diseases, or other health-related issues [14]. It encompasses a diverse range of interventions (single or in combination) and clinical disciplines, tailored to the specific needs of each individual. The ultimate goal of a rehabilitation process is to facilitate the highest level of independence and participation in daily life, school, work, and leisure activities, adapting to limitations when necessary and enhancing overall well-being. For instance, a person recovering from a low back injury might engage in a rehabilitation process that includes education on back care, targeted exercises, spinal manipulation, and psychological support to manage pain and promote re-engagement in daily activities, exemplifying a holistic approach to recovery.

Given the comprehensive nature of the rehabilitation process, our systematic review remains open to the inclusion of studies that might focus on specific aspects of the rehabilitation process, including those that emphasize pain relief as a primary outcome. Recognizing pain as a significant barrier to participation and engagement in rehabilitation activities, studies dedicated to understanding and managing pain are considered valuable. Effective pain management is not only critical for the immediate relief of symptoms but also plays a crucial role in enabling individuals to actively participate in their rehabilitation journey and achieve long-term goals of independence and improved quality of life. Such an approach is aligned with the World Health Organization's person-centered perspective on rehabilitation, which emphasizes addressing the most pressing needs of individuals undergoing rehabilitation, including pain management [11].

We included randomized controlled trials (RCT), cohort studies, case–control studies, and mixed-methods studies (quantitative component) for effectiveness and safety of interventions; qualitative and mixed-methods studies (qualitative component) for users’ experiences, preferences, expectations, and valued outcomes of interventions; and trial- and model-based full economic evaluations for cost-effectiveness of interventions (Table 2).

### Information sources

A health sciences librarian developed search strategies reviewed by a second health sciences librarian, using the Peer Review of Electronic Search Strategies (PRESS) checklist [15, 16]. The searches included a combination of subject headings specific to databases (e.g., MeSH in MEDLINE) and free text words to capture the key concepts of rehabilitative management of back pain in children and adolescents (Additional file 2).

We searched the following databases from inception to June 16, 2023: MEDLINE (Ovid), Embase (Ovid), PsycINFO (Ovid), CINAHL (EBSCO*host*), the Index to Chiropractic Literature (Chiropractic Library Collaboration), the Cochrane Controlled Register of Trials (Ovid), and EconLit (EBSCO*host*).

To mitigate the potential impact of publication bias, we further searched: 1) reference lists of included studies and relevant systematic reviews; 2) three websites (the Canadian Paediatric Society, the American Academy of Pediatrics, the European Paediatric Association); and 3) the WHO International Clinical Trials Registry Platform [17]. We included studies in any language.

### Screening for eligibility

We conducted training exercises prior to initiating the screening process. Reviewers screened a random sample of 50 titles/abstracts and 25 full-text articles. Paired reviewers reached ≥ 90% agreement before starting screening [18].

Pairs of reviewers independently screened titles and abstracts retrieved from electronic databases, and subsequently the full text of each selected article to confirm inclusion. Paired reviewers discussed disagreements to reach consensus, involving a third reviewer, if necessary.

Furthermore, one reviewer screened reference lists of included studies and relevant systematic reviews, the three websites, and protocols retrieved from the WHO International Clinical Trials Registry Platform. A second reviewer reviewed the screening performed by the first reviewer. Disagreements were resolved through discussion.

### Risk of bias in individual studies

We assessed the quality of studies using the Cochrane Risk of Bias (ROB) 1 tool [19] for RCTs; the risk of bias tool for nonrandomised studies for interventions (ROBINS-I) for cohort studies [20]; and the Joanna Briggs Institute (JBI) Critical Appraisal Checklist for qualitative studies [21]. We categorized the validity or credibility of each study as either low risk of bias, unclear or high risk of bias. Paired reviewers independently assessed the eligible studies for quality. We contacted one author to request additional data for clarification [22]. Any disagreements between reviewers were resolved through discussion or with a third reviewer.

### Data items and data extraction process

Paired reviewers independently extracted the data from all eligible studies and solved disagreements through discussion or a third reviewer. For the quantitative studies, we extracted data on the study and participant characteristics; intervention and comparator intervention characteristics using the Template for Intervention Description and Replication (TIDieR) checklist [23]; outcomes according to the ICF categories [24–26]; adverse events; key findings; and methodological quality. We used the PerSPecTIF question formulation framework to guide data extraction for the qualitative studies regarding the items: perspective, setting, phenomenon of interest, environment, timing, and findings (e.g., themes) [27].

### Data synthesis

We used a sequential approach at the review level to synthesize and integrate the data [28]. This involved separate quantitative and qualitative findings synthesis followed by integration of the resultant quantitative and qualitative evidence.

#### Quantitative synthesis

We assessed clinical heterogeneity among studies. Differences in populations, interventions, comparators, or outcomes across studies resulted in clinical heterogeneity.

To quantify the effectiveness of interventions, effect estimates (e.g., mean differences [MD], odds ratio or relative risk) and precision of the estimate (95% confidence interval [CI]) were extracted or computed. This systematic review used two criteria to determine whether an intervention was effective: 1) precision of the estimate and 2) magnitude of the estimate. Generally, differences were considered statistically significant if the 95% CI excluded zero in the mean difference (MD) or one in a risk ratio. An effect estimate of at least 10% of the range of the scale (for mean differences or median scores) or at least 10% difference for dichotomous outcomes, was considered clinically important [29]. We described the effectiveness of interventions as either “improve/reduce” or “make little difference” to outcomes in comparison to placebo/sham, control or another intervention (Table 3). An intervention was considered to “improve/reduce” outcomes (depending on direction) versus the comparison if the effect estimate was clinically important, and its 95% CI was statistically significant. An intervention was considered “make little difference” to outcomes versus the comparison if the effect estimate was: 1) not clinically important; or 2) the 95% CI was not statistically significant. We assessed the safety of interventions by identifying and categorizing adverse events reported in studies.

**Table 3:** Standard statements for reporting effects

	**Important benefit/harm**^**a**^	**No important benefit/harm**^**b**^
High certainty evidence	*[Intervention]* improves/reduces *[outcome]* (high certainty evidence)	*[Intervention]* makes little difference to *[outcome]* (high certainty evidence)
Moderate certainty evidence	*[Intervention]* probably improves/reduces *[outcome]* (moderate certainty evidence)	*[Intervention]* probably makes little difference to *[outcome]* (moderate certainty evidence)
Low certainty evidence	*[Intervention]* may improve/reduce *[outcome]* (low certainty evidence)	*[Intervention]* may make little difference to *[outcome]* (low certainty evidence)
Very low certainty evidence	It is uncertain whether *[intervention]* improves/reduces *[outcome]* because the certainty of this evidence is very low	

Adapted from: Cochrane Effective Practice and Organisation of Care (EPOC). Reporting the effects of an intervention in EPOC reviews. EPOC resources for review authors. 2018

^a^If an effect estimate was at least 10% of the range of the scale (for mean differences or median scores) or at least 10% difference for dichotomous outcomes, and the 95% CI did not cross the line of no effect, the effect was worded as the intervention “improve/reduce” on the outcome

^b^In all instances, if the 95% CI crossed the line of no effect, the effect was worded as the intervention having ‘little or no difference’ on the outcome versus describing a specific direction of effect (e.g., improve, reduce)

We applied the Grading of Recommendations, Assessment, Development, and Evaluation (GRADE) approach to assess the certainty of evidence for each outcome (Table 4) [30]. Recognizing the unique challenges of rehabilitation research, we adapted our application of GRADE to emphasize a context-sensitive analysis across five key domains: risk of bias, imprecision, publication bias, inconsistency, and indirectness, as suggested by Cancelliere et al. (2023) [31]:

- 1.Risk of Bias: We assessed the risk of bias in individual studies, understanding that the internal validity of studies is crucial for confidence in our findings. High-quality (low risk of bias) studies were prioritized to ensure the credibility of our evidence synthesis.
- 2.Imprecision: We evaluated the precision of effect estimates, paying close attention to the width of confidence intervals, while also taking into account minimal clinically important differences.
- 3.Publication Bias: To mitigate the potential for publication bias, we systematically searched for and included studies from a broad range of sources, including reference lists of included studies and relevant systematic reviews, targeted websites, and the World Health Organization International Clinical Trials Registry Platform.
- 4.Inconsistency: Given the expected challenge of achieving clinical homogeneity in context-sensitive research like ours, we anticipated findings from only one study per PICO question. Therefore, we did not automatically downgrade the certainty of evidence for inconsistency if only one study was available. We recognized the inherent heterogeneity of rehabilitation interventions and their outcomes, prompting us to judiciously evaluate the homogeneity (or lack thereof) among populations, interventions, comparators, and outcomes across studies before deciding on meta-analyses or opting for a descriptive synthesis approach when more appropriate.
- 5.Indirectness: We addressed indirectness by using clear and focused eligibility criteria to enhance the applicability of our findings to the target patient population. We ensured the evidence directly addressed our research question by confirming the direct relevance of populations, interventions, comparators, and outcomes to our clinical focus.

**Table 4:** Grading the evidence notes

Risk of bias
Options are not serious, serious (rate certainty of evidence down one level, e.g., from high to moderate), and very serious (rate certainty of evidence down two levels, e.g., from high to low): 1. Not serious: study rated as ‘low risk of bias’ or ‘unclear risk of bias’ (e.g., unclear co-interventions, no detailed randomization method described but similar baseline characteristics between groups) 2. Serious: study rated as ‘high risk of bias’ with unbalanced baseline characteristics between groups, unclear co-interventions, high/unbalanced drop-out and/or unclear intention-to-treat analysis 3. Very serious: study rated as ‘high risk of bias’ with unclear randomization sequence generation, inadequate allocation concealment and/or uncler/lack of blinding
Imprecision
Options are not serious, serious (rate certainty of evidence down one level), and very serious (rate certainty of evidence down two levels). Imprecision assessed using between-group effect [point estimate (95% CI)] 1. Not serious: If the point estimate is not clinically important: the upper and lower boundaries of the CI do not cross a clinically important threshold; the CI may cross the null as long as neither boundary crosses a clinically important threshold. If the point estimate is clinically important: the CI does not cross the null and the boundaries do not cross a clinically important threshold 2. Serious: If the point estimate is not clinically important: the CI may or may not cross the null but one of the boundaries crosses a clinically important threshold. If the point estimate is clinically important: the CI may cross the null but does not cross a clinically important threshold in the other direction 3. Very serious: If the point estimate is or is not clinically important: the CI crosses the boundaries of both appreciable harm and benefit (i.e., very wide CI)
Indirectness
Options are not serious, serious (rate certainty of evidence down one level), and very serious (rate certainty of evidence down two levels). Indirectness assessed whether the patients, interventions, or outcomes are different from the research question under investigation
Inconsistency
Options are not serious, serious (rate certainty of evidence down one level), and very serious (rate certainty of evidence down two levels). Inconsistency assessed effect estimate variance in direction or magnitude 1. Not serious: effect estimates are consistent in direction and magnitude across studies 2. Serious: effect estimates vary in magnitude across studies and the heterogeneity could not be explained 3. Very serious: effect estimates vary in direction across studies and the heterogeneity could not be explained
Publication bias
Publication bias assessed using funnel plot if possible, or based on available information from clinical trial registries

#### Integration of quantitative and qualitative evidence

We integrated the evidence by juxtaposing findings in a matrix to generate hypotheses regarding the effectiveness and safety of rehabilitation interventions for LBP in children and adolescents [28].

## Results

### Study selection

**Fig. 1:**
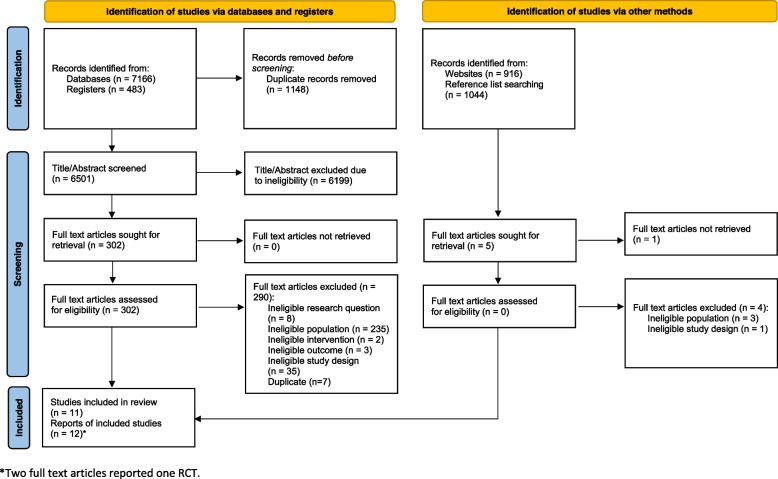
Identification and Selection of Articles (PRISMA 2020 flow diagram)

We screened 8461 citations and 307 full-text articles, and included 11 studies (Fig. 1). Of these 11 studies, the effectiveness of rehabilitation interventions were investigated in 10 quantitative studies including eight RCTs (518 participants) [22, 32–39] and two non-randomized clinical trials (40 participants) [40, 41] (Table 5), and one qualitative study investigated patients’ experience of physiotherapy (14 Participants) [42] (Table 6). We did not identify studies on cost-effectiveness of rehabilitation interventions. One RCT was reported in two full text articles [35, 36]. Two hundred ninety-five articles were excluded based on full text screening due to: 1) ineligible research question (8 articles); 2) ineligible population (238 articles); 3) ineligible intervention (2 articles); 4) ineligible outcome (3 articles); 5) ineligible study designs (36 articles); 6) duplicates (7 articles); and 7) cannot retrieve (1 article) (Additional file 3).

**Table 5:** Characteristics of Quantitative Studies

First Author, Country, Study Design	Year	Total participants	Clinical condition	Age (years)		Female sex No, (%)	Interventions	
				**Mean/ median**	**SD/ range**		**Intervention**	**Comparison**
***Spinal manipulation***								
Evans (United States) RCT [33]	2018	185	Adolescents (12–18 years) with nonspecific LBP with or without leg pain, VAS ≥ 3/10; subacute/recurrent (current episode 2–12 weeks duration with at least one similar episode in the past year) or chronic (current episode ≥ 12 weeks duration)	I: 15.5 C: 15.3	I: 1.6 C: 1.8	I: 65 (70%) C: 62 (67%)	**Type:** SMT + Exercise SMT: provided by chiropractors; techniques: high velocity low amplitude (preferred), low velocity low amplitude mobilization, flexion-distraction or drop-table assisted; up to a few minutes of ice/heat or soft tissue massage as needed **Duration:** 12 weeks **Frequency:** 1–2 x/week (20-min sessions); 8–16 sessions total **Setting:** clinic Exercise: provided by chiropractors or exercise therapists; self-care education; supervised sessions; components: aerobic, stretching, strengthening; home exercises accompanied by 20–40 min of aerobic activity **Duration:** 12 weeks **Frequency:** Supervised exercises: 1-2x/week (45-min sessions), 8–16 sessions total; Home exercises: 2x/week **Setting:** clinic/home	**Type:** Exercise (provided by chiropractors or exercise therapists; self-care education; supervised sessions; components: aerobic, stretching, strengthening; home exercises accompanied by 20–40 min of aerobic activity) **Duration:** 12 weeks **Frequency:** Supervised exercises: 1-2x/week (45-min sessions), 8–16 sessions total; Home exercises: 2x/week **Setting:** clinic/home
Selhorst (United States) RCT [22]	2015	35	Adolescents (13–17 years) with nonspecific LBP < 90 days duration	14.88	1.27	21 (62%)	**Type:** SMT + Exercise SMT: provided by physical therapists; technique: side-posture lumbar manipulation performed on symptomatic side **Duration:** 1 week **Frequency:** 2x/week **Setting:** Clinic Exercise: provided by physical therapist; components: lumbar stabilization, range of motion, postural training, core strengthening, stretching, addition of high-level functional exercises as indicated to promote return to activity **Duration:** 4 weeks **Frequency:** 2x/week **Setting:** Clinic	**Type:** Sham SMT + Exercise Sham SMT: provided by physical therapists; technique: patient side-lying, therapist passively flexed both hips to achieve slight lumbar flexion at patient’s most painful vertebral level, equal and opposite force applied to spinous process with both hands without inducing motion **Duration:** 1 week **Frequency:** 2x/week **Setting:** Clinic Exercise: provided by physical therapist; components: lumbar stabilization, range of motion, postural training, core strengthening, stretching, addition of high-level functional exercises as indicated to promote return to activity **Duration:** 4 weeks **Frequency:** 2x/week **Setting:** Clinic
***Group-based exercise***								
Fanucchi (South Africa) RCT [34]	2009	72	Children (12–13 years) with low back pain in the previous three months	12.3	0.7	I: 15 (38.5%) C: 18 (54.6%)	**Type:** Progressive exercise (instruction provided by PT; 10–15 min educational session about the importance of exercise, core musculature, posture and spinal alignment; weekly home exercise program including class-taught exercises; continuation of normal physical education classes, sports, and physical activity) **Duration:** 8 weeks **Frequency:** 1x/week (40–45 min sessions) **Setting:** School	**Type:** Control (no intervention; continuation of normal physical education classes, sports, and physical activity) **Duration:** 8 weeks **Frequency:** N/A **Setting:** N/A
Harringe (Sweden) Cohort study [40]	2007	*With and without LBP:* 51 (I: 33; C: 18) *With LBP:* 24 (I: 15; C: 4)	Female top level national gymnasts (11–16 years) with LBP (pain between the 12th rib and gluteal folds) more than 1 day during a 4-week baseline period	I: 13 C: 14	I: 11–15 C: 12–16	I: 15 (100%) C: 4 (100%)	**Type:** Specific segmental muscle control exercises (group training program provided by PT; abdominal hollowing with progression: prone, four-point kneeling, prone with diagonal elevation of arm and leg, standing on balance board, in a basic trampette jump; 10 repetitions using 10-s holds; pressure biofeedback unit used initially to ensure correct muscle contraction) **Duration:** 8 weeks **Frequency:** 3–4 times/week **Setting:** Gymnasium	**Type:** Control (visits provided by PT; given time for questions regarding injuries; provided advice and regime) **Duration:** 8 weeks **Frequency:** 3–4 times/week **Setting:** Gymnasium
Jones (1262 and 1267) (United Kingdom) RCT [35, 36]	2007	62	Adolescents (Grade 9 and 10 students) with recurrent nonspecific LBP as determined using a standardized questionnaire	I: 14.6 C: 14.6	I: 0.6 C: 0.5	Not reported	**Type:** Exercise rehabilitation (group-based; progressive program of strengthening and stabilization, range of motion, and aerobic exercises for the back and lower extremity; standardized with respect to number of exercises, repetitions, progression and schedule) **Duration:** 8 weeks **Frequency:** 2 x/week (30-min sessions) **Setting:** school (home exercise encouraged)	**Type:** Control (continue normal daily activities) **Duration:** 8 weeks **Frequency:** N/A **Setting:** N/A
Vitman (Israel) RCT [39]	2022	*33*	Children and adolescents (10 – 18 years) with LBP	I: not reported C: not reported	I: not reported C: not reported	Not reported	**Type:** Weekly physiotherapy + monthly physiotherapy and home exercise Weekly physiotherapy: 45-min group session with two physiotherapists, 21-exercise group therapy program **Duration:** 12 weeks **Frequency:** 1x/week **Setting:** Clinic Monthly physiotherapy and home exercise: same as the comparison group	**Type:** Monthly physiotherapy (i.e., personally-tailored comprehensive training) and home exercises Physiotherapy: 40-min session consisting of personally tailored training for muscle endurance, flexibility, and strength, as well as instructions on body awareness and application of biomechanical and ergonomic principles Home exercises: 5–6 individualized exercises, 1 set 10 repetitions (10–15 min/day). Diary kept of home practice to monitor **Duration:** 12 weeks **Frequency:** 1x/monthy **Setting:** Clinic
***Whole-body vibration***								
Jung (Korea) RCT [37]	2020	50	Adolescents (10 – 19 years) with LBP ≥ 3 months, VAS ≥ 3/10 and able to perform sit-to-stand movements without assistance	I: 18 C: 18	I: 0.65 C: 0.68	I: 10 (40%) C: 12 (48%)	**Type:** Whole-body vibration + trunk stabilization exercise Six exercises (squat, bridge, single bridge and knee flex, side bridge, plank) performed on whole-body vibration machine (15 Hz and 2 mm amplitude). Exercise duration was 60 s for single bridge, bridge and knee flex, and plank or 90 secs for squat, bridge, side bridge, performed for 2 sets with 30 s break in between **Duration:** 12 weeks **Frequency:** 3x/week **Setting:** Clinic (supervised by physiotherapist)	**Type:** Trunk stabilization exercise Six exercises (squat, bridge, single bridge and knee flex, side bridge, plank). Exercise duration was 60 s for single bridge, bridge and knee flex, and plank or 90 secs for squat, bridge, side bridge, performed for 2 sets with 30 s break in between **Duration:** 12 weeks **Frequency:** 3x/week **Setting:** Clinic (supervised by physiotherapist)
***Cognitive functional therapy***								
Ng (Australia) RCT [38]	2015	36	Adolescent male rowers (14–19 years) with nonspecific LBP, VAS > 3/10	I: 16.3 C: 15.2	I: 1.5 C: 1.5	0 (0%)	**Type:** Cognitive functional approach (provided by a physiotherapist; components: education, discussion about factors contributing to back pain, movement training and body awareness, functional integration, conditioning) **Duration:** 8 weeks **Frequency:** 1x/week for first two weeks, 1x/2 weeks for remainder (1 h initial; 30-min subsequent); total 5 sessions **Setting:** local rowing club or university laboratory	**Type:** Control (no intervention; free to seek treatment from other providers)
***Multimodal care***								
Ahlqwist (Sweden) RCT [32]	*2008*	45	Adolescents (12–18 years) with nonspecific LBP (lumbar pain in a defined area); referred by a physician or nurse; VAS > 2/10	I: 15 C: 14	I: 13–18 C: 12–17	I: 15 (65%) C: 16 (73%)	**Type:** Individualized physical therapy and exercise + standardized home exercise + education Individualized physical therapy and exercise: exercises supervised by a physical therapist (15 reps/exercise; general and specific exercises including conditioning, active and passive mobility, strengthening and coordination; resistance gradually increased); individualized therapy (manual therapy, mechanical diagnostic therapy) **Duration:** 12 weeks **Frequency:** 1x/week **Setting:** clinic Standardized home exercise: body weight for resistance; 2 sets of 10 reps/exercise **Duration:** 12 weeks **Frequency:** 2x/week **Setting:** home Education: functional anatomy, ergonomics, pain management **Frequency:** 1 session **Setting:** clinic	**Type:** standardized home exercise + education Self-training: conditioning exercises (brisk walks, jogging, bicycling, swimming) **Duration:** 12 weeks **Frequency:** 3x/week **Setting:** home; follow-up in clinic at 1 week; follow-up by telephone at 6 weeks Standardized home exercise: body weight for resistance; 2 sets of 10 reps/exercise **Duration:** 12 weeks **Frequency:** 3x/week **Setting:** home Education: functional anatomy, ergonomics, pain management **Frequency:** 1 session **Setting:** clinic
Selhorst (United States) Cohort study [41]	2021	16	Adolescent (12 – 19 years) athletes (participating in sport activity ≥ 2 times/week prior to the onset of LBP) who reports acute LBP (< 3 months) that increases during lumbar extension	I: 14.5 C: 15.5	I: 12.1 C: 1.4	I: 5 (62%) C: 3 (38%)	**Type:** Physical therapist guided functional progression program (PT First) No advanced imaging was obtained at the beginning of the treatment PT First Program 3-phase program: Participants were on rest from their sport Phase I: core strengthening in neutral spine, directional preference if identified, hip strengthening, peri-scapular strengthening, flexibility exercises, manual therapy as needed, modalities for pain (sparingly) Phase II: core strengthening in functional range, hip and peri-scapular strengthening, flexibility exercises, manual therapy (sparingly), light running, jumping Phase III: Return to sport activity with focus on functional return to all aspects of sport Patients who fail to progress after 5 weeks either were treated as a presumed spondylolysis or had advanced imaging. They received two months of rest except for daily activities and home exercise program, following this, they completed physical therapy before returning to sport **Duration:** Variable **Frequency:** 2x/week **Setting:** Clinic	**Type:** Biomedical model Advanced imaging was obtained to diagnose the injury and participants diagnosed with non-specific LBP or spondylolisthesis Patients with non-specific LBP: physical therapy and progressed to sport immediately Patients with a bony or spondylolytic injury: preliminary 2–3 month rest from activity, bracing if indicated, followed by 4–6 weeks of physical therapy. Physical therapy was individualized based on patient’s presentation Physical therapy: **Duration:** 4–6 weeks **Frequency:** 2x/week **Setting:** Clinic

*Abbreviations*: *C* Comparison, *I* Intervention, *LBP* Low Back Pain, *RCT* randomized controlled trial, *VAS* Visual Analog Scale

**Table 6:** Evidence profile of the included qualitative study(42)

Perspective	Setting	Phenomenon of interest	Environment	Time/timing	Findings
From perspectives of male and female adolescents (12–18 years old) with low back pain	Clinic	Individually tailored physical therapy and home exercise	the Gothenburg area, Sweden	12 weeks	Mobilizing own resources in successfully gaining body confidence in daily life: 1. Coaching from the physiotherapist: professional support; being aware of inherent capabilities; and trust in the physiotherapist and hope of recovery. Participants appreciated the attitude and professionalism of the physiotherapist 2. This theme consists of three subcategories: information from the school nurse; information from the physiotherapist; insight and reorientation of back pain that relates to participants’ understanding and leads to change 3. Compliance with physiotherapy: exercises provide structure; gaining energy from treatment; gaining confidence in exercises. Tailored exercises with the physiotherapist restored control to participants on physical as well as psychological levels 4. Gaining energy from pain-free moments: handling pain; ability to achieve change; distraction by recovery. Participants experienced a growing awareness of their bodies and their pain, as well as an increased easing of tension and a certain pain relief

### Study characteristics

#### *Quantitative studies *(Table 5)

The mean age of participants ranged from 12.3 to 18 years old. Among participants in the eight studies reporting sex, 50.4% (*n*= 261) were female [22, 32–34, 37, 38, 40, 41]. All studies included participants with nonspecific LBP, with no studies focusing on TSP. Participants received various rehabilitation interventions including exercise (4 studies) [34–36, 39, 40], spinal manipulation (2 studies) [22, 33], cognitive therapy (1 study) [38], whole-body vibration (1 study) [37], and multimodal care (2 studies) [32, 41]. The duration of rehabilitation interventions varied: 1) four weeks (1 study) [22]; 2) eight weeks (4 studies) [34–36, 38, 40]; and 3) 12 weeks (4 studies) [32, 33, 37, 39]; and 4) variable duration (1 study) [41]. These 10 studies investigated rehabilitation interventions: 1) as an addition to active comparison interventions where the attributable effect of the comparison interventions can be isolated (4 studies) [32, 33, 37, 39]; 2) compared to no treatment (3 studies) [34–36, 38]; 3) compared to sham (1 study) [22]; and 4) compared to other active interventions (2 studies) [40, 41], respectively. Outcomes included LBP intensity (9 studies) [22, 32–40], function (5 studies) [22, 32, 33, 38, 41], quality of life (2 studies) [32, 33], improvement (2 studies) [22, 33], satisfaction (1 study) [33], wellbeing (1 study) [34], feelings about school and life (1 study) [34], absence from school or physical activity (1 study) [35], and health resource utilization (1 study) [22]. The 10 studies were clinically heterogeneous, therefore, a meta-analysis was not conducted [43].

#### *Qualitative study *(Table 6)

The qualitative study used grounded theory methodology to explore the experience of adolescents (aged 12–18 years) with LBP who received individually tailored physical therapy and home exercise [42].

### Risk of bias assessment

Among eight RCTs, one was rated as overall unclear risk of bias [33] and seven were rated as high risk of bias [22, 32, 34–39] (Table 7, Risk of Bias Assessment of Included Studies). The two non-randomized clinical trials were rated as overall serious risk of bias (Table 8, Risk of Bias Assessment of Included Studies) [40, 41]. One qualitative study was rated as overall low risk of bias (Table 9) [42].

**Table 7:** Risk of Bias of Randomized Controlled Trials Based on the ROB 1 Tool Criteria

Study	Selection bias		Performance bias		Detection bias	Attrition bias		Reporting bias	Selection bias	Other bias				Over all risk of bias
	**Method of randomization**	**Treatment allocation concealed**	**Patient blinded to the intervention**	**Care provider blinded to the intervention**	**Outcome assessor blinded to the intervention**	**Drop-out rate**	**Intention to treat analysis**	**Free from selective outcome reporting**	**Similarity of group baseline characteristics**	**Co-interventions avoided or comparable**	**Compliance acceptable in all groups**	**Timing of outcome assessment similar**	**outcome measurement tools, conflicts of interest and funding**	
Ahlqwist et al. (2008) [32]	Low	Low	Unclear	Unclear	Unclear	Unclear	Unclear	Low	High	Unclear	Unclear	Low	Low	High
Evans et al. (2018) [33]	Low	Low	Unclear	Unclear	Unclear	12 weeks: SMT + ET: 1/93 (1.1%) ET: 4/92 (4.3%)	Low	Low	Low	Low	Low	Low	Low	Unclear
Fanucci et al. (2009) [34]	Low	Low	High	High	High	3 months: Exercise: 0%; No treatment: 3%	Unclear	Low	Unclear	Unclear	Low	Low	Low	High
Jones et al. (2007) [35, 36]	Unclear	Low	High	High	High	Control: 13% Exercise: 13%	High	Low	Unclear	Unclear	Low	Low	Unclear	High
Jung et al. (2020) [37]	Low	Unclear	Unclear	Unclear	Unclear	Unclear	Unclear	Unclear	Unclear	Unclear	Unclear	Low	Unclear	High
Ng et al. (2015) [38]	Low	Low	High	Low	High	8 weeks: Cognitive functional approach: 11.8% Active control: 5.3% 12 weeks: Cognitive functional approach: 11.8% Active control: 5.3%	Unclear	Low	Low	Unclear	Low	Low	Low	High
Selhorst et al. (2015) [22]	Low	Unclear	Unclear	Low	Unclear	4 weeks (PSFS, NPRS): Sham + Exercise: 35.2% Manipulation + Exercise: 22.2% 6 months (chronic symptoms, recurrence of symptoms, additional treatment): Sham + Exercise: 11.8% Manipulation + Exercise: 5.6%	Low	Low	Unclear	Unclear	Unclear	Low	Low	High
Vitman et al. (2022) [39]	Unclear	Unclear	Unclear	Unclear	Unclear	Total drop-out: 7.4%. Unclear about the group allocation of the drop-outs	High	Low	Unclear	Unclear	Unclear	Low	Low	High

**Table 8:** Risk of Bias of Cohort Studies Based on the ROBIS-I tool Criteria

Study	Bias due to confounding	Bias in selection of participants into the study	Bias in classification of interventions	Bias due to deviations from intended interventions	Bias due to missing data	Bias in measurement of outcomes	Bias in selection of the reported result	Overall risk of bias
Harringe et al. (2007) [40]	Serious	Low	Low	Low	Serious	Serious	Low	Serious
Selhorst et al. (2021) [41]	Serious	Low	Low	Low	Low	Serious	Low	Serious

**Table 9:** Risk of Bias of Qualitative Study Based on the JBI tool Criteria

Ahlqwist et al. (2012) [42] (Ahlqwist and Sällfors 2012) [42]	
1.Is there congruity between the stated philosophical perspective and the research methodology?	No
2.Is there congruity between the research methodology and the research question or objectives?	Yes
3.Is there congruity between the research methodology and the methods used to collect data?	Yes
4.Is there congruity between the research methodology and the representation and analysis of data?	Yes
5.Is there congruity between the research methodology and the interpretation of results?	Yes
6.Is there a statement locating the researcher culturally or theoretically?	No
7.Is the influence of the researcher on the research, and vice- versa, addressed?	Yes
8.Are participants, and their voices, adequately represented?	No
9.Is the research ethical according to current criteria or, for recent studies, and is there evidence of ethical approval by an appropriate body?	Yes
10.Do the conclusions drawn in the research report flow from the analysis, or interpretation, of the data?	No
Overall appraisal	Include

### Synthesis of quantitative studies

#### Spinal manipulation

Two RCTs evaluated the effectiveness of spinal manipulation in adolescents with LBP [22, 33]. (Table 10).

**Table 10:** Brief Evidence Profile

Intervention	Overall findings
***Spinal manipulation***	
Spinal manipulation and exercise versus Same exercise (1 RCT) (Evans et al., 2018) [33]	Spinal manipulation (1–2 sessions/week) over 12 weeks • **Reduce** pain intensity (low to moderate certainty evidence) • Do **not** provide additional benefit in improving function, quality of life, patient-reported improvement and patient-reported satisfaction (moderate certainty evidence) • Do **not** cause more adverse events than control (very low certainty evidence)
Spinal manipulation and exercise versus Sham and same exercise (1 RCT) (Selhorst et al., 2015) [22]	Spinal manipulation (2 sessions in total over one week) does **not** bring additional benefits in improving • Pain intensity (low to moderate certainty evidence) • Function (low certainty evidence) • Improvement (low certainty evidence) • Recurrence of symptoms (very low certainty evidence) • Health resources use (very low certainty evidence) And • And do **not** cause more adverse events than control (very low certainty evidence)
***Group-based exercise***	
Group-based exercise, monthly personal tailored exercise and home-based exercise versus Monthly personal tailored exercise and home-based exercise (1 RCT) (Vitman et al., 2022) [39]	Group-based exercise (one session/week over 12 weeks) • Do **not** reduce LBP intensity (very low certainty evidence)
Group-based exercise versus No treatment (2 RCTs) (Fanucchi et al., 2009 [34]; M. Jones et al.; 2007 [35], M. A. Jones et al., 2007) [36]	Group-based progressive exercise provided at school for eight weeks • **Reduce** pain intensity (very low to low certainty evidence) ((Fanucchi et al., 2009 [34]; M. Jones et al.; 2007 [35], M. A. Jones et al., 2007) [36] • Do **not** improve absence from physical activity and school (very low to low certainty evidence) (M. Jones et al., 2007 [35]; M A. Jones et al., 2007) [36] • Do **not** improve well-being and feelings about school and life (very low to low certainty evidence) (Fanucchi et al., 2009) [34]
Group-based exercise versus Advice and individual training (1 non-randomized controlled trial) (Harringe et al., 2007) [40]	Group-based muscle control exercise over eight weeks • **Reduce** days with pain (very low certainty evidence) • Do **not** reduce maximum and median pain intensity (very low certainty evidence)
***Whole-body vibration***	
Whole-body vibration and trunk stabilization exercise versus Trunk stabilization exercise (1 RCT) (Jung et al., 2020) [37]	Whole-body vibration (3 times per week over 12 weeks) • Do **not** reduce LBP intensity (very low certainty evidence)
***Cognitive functional therapy***	
Cognitive functional therapy Versus No treatment (1 RCT) (Ng et al., 2015) [38]	Cognitive functional therapy over eight weeks • **Reduce** LBP intensity (very low certainty evidence) • **Improve** function (very low certainty evidence)
***Multimodal care***	
Multimodal care, home exercise and education Versus Home exercise and education (1 RCT) (Ahlqwist et al., 2008) [32]	multimodal care (including supervised exercise; manual therapy and mechanical diagnostic therapy as needed) (1 session per week over 12 weeks) does **not** provided additional benefit in • Reducing pain intensity (very low certainty evidence) • Improving function (low certainty evidence) • Improving quality of life (very low certainty evidence)
Physiotherapist-led multimodal care (exercise, manual therapy, modalities for pain) Versus Physician-led care (including physiotherapy) (1 non-randomized controlled trial) (Selhorst et al., 2021) [41]	Physiotherapist-led care (exercise, manual therapy, modalities for pain) • Do **not** improve function (very low certainty evidence)

*LBP* low back pain

##### Spinal manipulation and exercise versus same exercise

One RCT compared spinal manipulation (1–2 sessions/week over 12 weeks) plus exercise (12 weeks) to the same exercise [33].

For pain, immediately following a 12-week treatment, participants in the spinal manipulation group were more likely to experience a clinically important reduction (RR 2.15 [1.16, 3.98] for 75% pain reduction, moderate certainty evidence; and RR 2.68 [1.01, 7.12] for 100% pain reduction, low certainty evidence). Similar results were observed immediately and at 3 and 9 months following the 12-week treatment, with the largest effect size at 3 months and smallest at 9 months following the treatment. For details, see Table 10 and Additional file 4.

Twelve-week spinal manipulation made little difference to function (RMDQ, MD 0.54 [-0.25, 1.34]), quality of life (PedsQL, MD 1.33 [-1.64, 4.31]), patient-reported improvement (a 9-point scale, MD -0.29 [-0.66, 0.09]) or satisfaction (a 7-point scale, MD -0.36 [-0.65, -0.07]) immediately following the treatment (moderate certainty evidence). Similar results were observed at 3 months or 9 months following the treatment (moderate certainty evidence). For details, see Table 10 and Additional file 4.

Due to very low certainty evidence, it is uncertain whether participants in spinal manipulation plus exercise group and exercise alone group had similar chance of experiencing adverse events (RR 1.00 [0.16, 6.30]).

##### Spinal manipulation and exercise versus sham manipulation and same exercise

One RCT compared spinal manipulation (2 sessions in total over one week) and exercise (4 weeks) to sham spinal manipulation and the same exercise [22].

For pain, two sessions of spinal manipulation made little difference to LBP intensity (measured by scale 0–10 on numerical rating scale [NRS]) immediately following the treatment (MD -0.58 [-1.49, 0.33]) and at 5 months following the treatment (MD -0.26 [-0.82, 0.31]) (low to moderate certainty evidence).

Low certainty evidence suggests that spinal manipulation made little difference to function (PSFS) immediately following the treatment (MD 2.8 [-0.91, 5.51]) and at the 5 months following the treatment (MD 1.08 [-2.2, 4.36], PFPS), and improvement (Global Rating of Change) (MD 0.66 [-0.95, 2.27]) immediately following the treatment.

Due to very low certainty evidence, it is uncertain whether participants in both the spinal manipulation and sham groups were equally likely to use health resources (RR 0.59 [0.25, 1.39], by evaluation of seeking additional treatment for LBP during follow-up period) or have a recurrence of symptoms (RR 0.77 [0.45, 1.30], significant enough to impair participation during follow-up period).

Due to very low certainty evidence, it is uncertain whether participants in the spinal manipulation group and sham group had an equal chance of experiencing adverse events [22].

#### Group-based exercise

Four RCTs evaluated the effectiveness of group-based exercise in adolescents with LBP [34–36, 39, 40]. (Table 10, Additional file 4).

##### Group-based exercise, monthly personal tailored exercise and home-based exercise versus monthly personal tailored exercise and home-based exercise

One RCT compared group-based exercise (one session/week over 12 weeks) combined with monthly personal tailored exercise and home-based exercise to the same monthly exercise and home-based exercise [39]. It is uncertain whether the addition of weekly group exercise made little difference to LBP intensity (MD -1.2 [-2.65, 0.25], scale range 0–10 on a Visual Analogue Scale [VAS]; very low certainty evidence) immediately following the 12-week treatment.

##### Group-based exercise versus no treatment

Two RCTs compared group-based progressive exercise provided at school for eight weeks to no treatment34-36.

For pain, low certainty evidence suggests that group-based exercise reduced pain immediately following the treatment (MD -2.3 [-3.1 to -1.5]; scale range 0 to 10 on NRS; 1 RCT) [35, 36]. However, at 4 weeks following the treatment, it is uncertain whether group-based exercise reduced pain (MD -1.50 [-2.68, -0.32]; scale range 0 to 10 on VAS), or whether participants in group-based exercise group were less likely to have LBP (RR 0.74 [0.57, 0.94]; 1 RCT) [34]. Low certainty evidence from the same study suggests that group-based exercise participants were less likely to have LBP at 4 months following the treatment (RR 0.52 [0.34, 0.78]; 1 RCT) [34].

For absence from school, group-based exercise made little difference to absence from school during the past seven days (MD 0 [-0.1, 0.1]; low certainty evidence, 1 RCT) immediately following the treatment [35, 36]. For absence from physical activity, due to very low certainty evidence (1 RCT), it is uncertain whether group-based exercise made little difference to absence from physical activity during the past seven days (MD 0.6 day [-1, 0.2]) immediately following the treatment [35, 36].

For well-being, group-based exercise made little difference at 4 weeks following the treatment (MD 0 [-1.69, 1.69], scale range 5–30 on the Mental Health Inventory-5 (MHI-5), 30 = psychosocial well-being; 1 RCT, low certainty evidence) [34]. Similar results were observed for well-being, and feelings about school and life at 4 weeks and 4 months following the 8-week group exercise. For details, see Table 10 and Additional file 4.

##### Group-based exercise versus advice and individual training

One non-randomized clinical trial compared 8-week group-based muscle control exercise to advice and individual training [40]. Due to very low certainty evidence, it is uncertain whether group-based exercise reduced days with pain during the four weeks period immediately after the 8-week treatment (between-group mean difference not reported). Due to very low certainty evidence, it is uncertain whether group-based exercise made little difference to maximum and median pain intensity during the four weeks after the 8-week treatment (between-group mean difference not reported).

#### Whole-body vibration

##### Whole-body vibration and trunk stabilization exercise versus trunk stabilization exercise

One RCT evaluated whole-body vibration when added to trunk stabilization exercise in adolescents with LBP, it is uncertain whether its addition made little difference to LBP intensity when compared to trunk muscle stabilization exercise alone (MD -0.66 [-1.27, -0.05], scale range 0–10 on NRS; very low certainty evidence) immediately following a 12-week treatment among adolescents [37]. (Table 10, Additional file 4).

#### Cognitive functional therapy

##### Cognitive functional therapy versus no treatment

One RCT compared cognitive functional therapy to no treatment in adolescents with LBP [38]. Due to very low certainty evidence, it is uncertain whether 8 weeks of cognitive functional therapy 1) reduced LBP intensity following a 15-min ergometer trial immediately post-intervention (MD -2.4 [-4.1, -0.63], scale range 0 to 10 on NRS); and 2) improved function immediately following the treatment (MD 4.1 [0.9, 7.3], scale range 0–30 on PFPS, 30 = no function limitation) and four weeks after the 8-week treatment (MD 4.0 [0.8, 7.2], PFPS). (Table 10, Additional file 4).

#### Multimodal care

One RCT and one non-randomized controlled trial evaluated the effectiveness of multimodal care in adolescents with LBP [32, 41]. (Table 10, Additional file 4).

##### Multimodal care, home exercise and education versus home exercise and education

One RCT compared multimodal care (including supervised exercise; manual therapy and mechanical diagnostic therapy as needed) plus home exercise and education to the same home exercise and education [32].

Due to very low certainty, it is uncertain whether the addition of multimodal care made little difference to LBP intensity (MD -0.5 [-3.9, 2.9], scale range 0–10 on VAS) and quality of life measured by Child Health Questionnaire-Child Form (no standard deviation or 95%CI reported) when compared to home exercise and education alone immediately following a 12-week treatment.

For function, multimodal care did not improve function when compared to home exercise and education alone (MD -0.8 (-2.31, 0.7), scale range 0–24 on RMDQ) immediately following a 12-week treatment (low certainty evidence).

##### Physiotherapist-led multimodal care (exercise, manual therapy, modalities for pain) versus physician-led care

One non-randomized clinical trial compared physiotherapist-led care (exercise, manual therapy, modalities for pain) to physician-led care (including physiotherapy). The mean days of care provided was 62 days. It is uncertain whether multimodal care made little difference to function (MD 6 [-13.22, 25.22], scale range 0–100 on Micheli Functional Scale; very low certainty evidence) [41].

## Summary of quantitative findings

There is low to moderate certainty evidence that spinal manipulation (1–2 sessions/week over 12 weeks) and exercise may be associated with a greater likelihood of experiencing clinically important pain reduction versus exercise alone immediately following the intervention and in the short-term in adolescents with LBP [33]. There is very low to low certainty evidence that group-based exercise programs (over 8 weeks) may reduce pain immediately post-intervention and in the short-term in adolescents with LBP [34–36, 40]. Due to very low certainty evidence, the clinical benefit of cognitive functional therapy in improving pain and function is uncertain [38]. The three interventions included an education component and reasonable compliance of the interventions was achieved.

There is low certainty evidence that multimodal care (including supervised exercise; manual therapy and mechanical diagnostic therapy as needed) may not bring additional benefit in improving function when added to home exercise and education [32]. It is uncertain whether the addition of whole-body vibration made little difference to pain intensity when compared to trunk muscle stabilization exercise alone (very low certainty evidence) [37].

## Findings of qualitative study

The qualitative study explored the experiences of adolescents with LBP treated by individually tailored physical therapy and home exercise [42]. A core category, mobilizing own resources, emerged from the analysis, describing how adolescents with LBP succeed in managing their main concern, gaining body confidence, in daily life. The core category was divided into four categories labelled: 1) coaching from the physiotherapist, 2) seeking for information, 3) compliance with physiotherapy, and 4) gaining energy from pain-free moments (Table 6). Information-seeking is related to information available to participants that enhance their understanding and leads to change. Compliance with treatment refers to tailored exercises with the physiotherapist restoring control to participants on physical as well as psychological levels, which prompted them to return for the next appointment.

## Integration of quantitative and qualitative evidence

We used a joint display table to illustrate the connection between quantitative and qualitative results (Table 11). The interventions evaluated in four quantitative studies fulfilled subcategories 2 and 3 (i.e., seeking for information, compliance with treatment) (Table 11) [33, 34, 38, 40]. These interventions improved pain intensity and/or function (very low to moderate certainty evidence). Interventions evaluated in other six quantitative studies did not fulfill at least one of the two subcategories [22, 32, 35–37, 39, 41, 42]. All these interventions except one (group-based exercise) [35, 36] did not bring benefit or additional benefit if combined with other interventions.

**Table 11:** Presence of qualitative themes in quantitative studies

Theme	Ahlqwist 2008 Sweden [32]	Evans 2018 United States [33]	Fanucchi 2009 South Africa [34]	Harringe 2007 Sweden [40]	Jones 2007 United Kingdom [35, 36]	Jung 2020 Korea [37]	Ng 2015 Australia [38]	Selhorst 2015 United States [22]	Selhorst 2021 United States [41]	Vitman 2022 [39] Israel	Theme description
Coaching from care providers	NI	NI	NI	NI	NI	NI	NI	NI	NI	NI	This theme consists of three subcategories: professional support; being aware of inherent capabilities; and trust in the physiotherapist and hope of recovery. Participants appreciated the attitude and professionalism of the physiotherapist
Seeking for information	√	√	√	√	?	?	√	?	?	√	This theme consists of three subcategories: information from the school nurse; information from the physiotherapist; insight and reorientation of back pain that relates to participants’ understanding and leads to change
Compliance with treatment	?	√	√	√	√	?	√	?	√	?	This theme consists of three subcategories: exercises provide structure; gaining energy from treatment; gaining confidence in exercises. Tailored exercises with the physiotherapist restored control to participants on physical as well as psychological levels
Gaining energy from pain-free moments	NR	NR	NR	NR	NR	NR	NR	NR	NR	NR	This theme consists of three subcategories: handling pain; ability to achieve change; distraction by recovery. Participants experienced a growing awareness of their bodies and their pain, as well as an increased easing of tension and a certain pain relief
Overall risk of bias	High	Unclear	High	High	High	High	High	High	High	High	

?unclear (limited information and cannot make a judgement); √ acceptable compliance

*NI* no information, *NR* not directly related to the treatment

## Discussion

There is evidence of clinical benefit from spinal manipulation (low to moderate certainty) and group-based exercise (very low to low certainty). Multimodal care did not appear to be beneficial (very low to low certainty), and the benefit of both cognitive therapy and whole-body vibration were uncertain (very low certainty). Qualitative findings suggest that seeking/receiving information and compliance with treatment are important factors to mobilize own resources to manage in daily life.

Only two studies evaluated adverse events [22, 33]. Due to very low certainty evidence, it is uncertain whether participants receiving spinal manipulation and participants without spinal manipulation had similar likelihood of adverse events.

We identified neither studies on TSP, mixed methods studies, nor economic studies in children and adolescents.

### Strengths and limitations

This systematic review has strengths. First, this review included comprehensive and peer-reviewed literature search strategies and examined all non-surgical rehabilitation interventions without language restrictions. Second, this review used a definition of rehabilitation as proposed by Cochrane Rehabilitation, which allowed us to capture what can be considered components of broader rehabilitation interventions that are provided within the rehabilitation process.

Due to limited number of relevant studies and clinical heterogeneity, a meta-analysis could not be conducted. Second, it was challenging to apply GRADE to the included studies to examine rehabilitation interventions due to clinical heterogeneity and blinding issues caused by the nature of rehabilitation interventions. Therefore, we adapted the GRADE approach and tailored it to rehabilitation studies.

### Comparison to other systematic reviews and guideline

There are three systematic reviews identified in evaluating rehabilitation interventions for the management of LBP in pediatric population published between 2014 and 2019 [6–8]. Our review agreed with the systematic review by Michaleff et al. (2014) [8] suggesting that a supervised exercise program was better than no treatment. We augmented this conclusion by adding one non-randomized clinical trial [40]. However, we do not agree that a supervised exercise program reduces absences from physical activity due to a non-clinical important change [35, 36]. The systematic review by Driehuis et al. (2019) [6] suggested spinal manipulation did not bring adverse events; however, our review found the certainty of evidence is very low. Further studies are needed before making a conclusion about adverse events associated with spinal manipulation. Driehuis et al. did not identify studies on the effectiveness of spinal manipulation for LBP. Last, both Prevost et al. (2019) [7] and our review found that spinal manipulation reduced LBP intensity. Our review further clarified that spinal manipulation (1–2 sessions/week over 12 weeks) combined with exercise is probably associated with a greater likelihood of experiencing clinically important pain reduction immediately following the intervention and over the short-term versus exercise alone [33]. In addition to all the three reviews, we identified studies on the effectiveness of cognitive functional therapy, whole-body vibration and multimodal care.

### Implications

The findings in our review have important implications for clinical practice. First, as evident in the included qualitative study, the patient-doctor relationship should be highlighted, providing a foundation for a positive interaction that may facilitate increased compliance with treatment towards the goal of recovery (i.e., subcategories 2 and 3: coaching from care providers, compliance with treatment) [42]. Second, information provided via education/advice should be considered as part of care program [44]. Third, spinal manipulation and group-based exercise may be considered through shared decision-making to reduce pain intensity in adolescents with LBP based on low to moderate certainty evidence [33–36].

Compared to previous reviews, evidence is expanding. However, future studies with rigorous methodological quality are still needed. Two previous systematic reviews only identified four studies (including one case series and one cohort study without a control group) [7] and one study [6], respectively. Our review identified 10 quantitative studies (i.e., 8 RCTs, 2 non-randomized clinical trials) and one qualitative study. Of those, nine quantitative studies had high risk of bias. Specifically, blinding of participants, treatment providers and outcome assessors (e.g., participants for self-reported outcomes) are the main challenge in the included RCTs. This challenge is due to the nature of rehabilitation interventions. To minimize potential biases caused by these issues, future RCTs can be restricted to participants who are naïve to the studies interventions [31]. Alternatively, future RCTs can consider measuring treatment credibility/expectancy and blinding, and consider these in the analysis and interpretation of potential biases and the implications on intervention effect estimates [31]. Furthermore, apart from RCTs, future studies can consider various study designs (e.g., quasi-experimental design, qualitative, mixed methods, and implementation studies) depending on the research question [31]. These study designs can complement the evidence obtained from RCTs, therefore contributing to a more holistic perspective on the evaluation of benefits and harms, specifically for a context-sensitive condition (e.g., LBP). For example, qualitative studies can explore patients’ lived experiences and assist better understanding of evidence from RCTs regarding treatment effects, compliance etc.

## Conclusion

Spinal manipulation and group-based exercise may be beneficial in reducing LBP intensity in adolescents based on evidence ranging from very low to moderate certainty. Education should be provided as part of a care program. Studies with rigorous methodological quality are needed.

### Supplementary Information

Additional file 1: PRISMA 2020 Checklist. Preferred Reporting Items for Systematic Review and Meta-Analysis.

Additional file 2: Literature search strategy.

Additional file 3: Exclusion reasons for studies excluded in full text screening.

Additional file 4: Evidence Profile.

## Data Availability

All data generated/analysed in this systematic review are included in this published systematic review and its supplementary information files.

## Abbreviations

95%CI: 95% Confidence interval
JBI critical appraisal checklist: The Joanna Briggs Institute Critical Appraisal Checklist
LBP: Low back pain
MD: Mean difference
MHI-5: Mental Health Inventory-5
NRS: Numerical rating scale
PedsQL: Pediatric Quality of Life Inventory
PRESS: The Peer Review of Electronic Search Strategies
PRISMA: The Preferred Reporting Items for Systematic Reviews and Meta-Analyses
PROSPERO: The International Prospective Register of Systematic Reviews
PSFS: Patient Specific Functional Scale
RCT: Randomized controlled trial
RMDQ: Roland Morris Disability Questionnaire
ROB 1 tool: The Cochrane Risk of Bias 1 tool
ROBINS-I: The risk of bias tool for nonrandomised studies for interventions
RR: Relative risk
SWiM: The Synthesis Without Meta-analysis
TIDieR: The Template for Intervention Description and Replication checklist
TSP: Thoracic spine pain
VAS: Visual analog scale
