# Supplementary material for: Rehabilitation of back pain in the pediatric population: a mixed studies systematic review
Source: Chiropr Man Therap. 2024 May 8;32:14. doi: 10.1186/s12998-024-00538-z (PMC11080233; doi:10.1186/s12998-024-00538-z)
Supplement: Supplementary file 2 — Additional file 2: Literature search strategy. [file 12998_2024_538_MOESM2_ESM.docx]

**Additional file 2. MEDLINE (OVID) search strategy**

| 1 | exp Infant/ |
| --- | --- |
| 2 | Child, Preschool/ |
| 3 | Child/ |
| 4 | Adolescent/ |
| 5 | Pediatrics/ |
| 6 | (baby or babies).ab,ti. |
| 7 | "newborn*".ab,ti. |
| 8 | (infant or infants).ab,ti. |
| 9 | (child or children*).ab,ti. |
| 10 | (adolescent* or adolescence).ab,ti. |
| 11 | (teen or teens or teenager).ab,ti. |
| 12 | (pediatric* or paediatric*).ab,ti. |
| 13 | (young adj3 (person* or people)).ab,ti. |
| 14 | emerging adult*.ab,ti. |
| 15 | "youth*".ab,ti. |
| 16 | or/1-15 [**pediatric population] |
| 17 | exp Back Injuries/ |
| 18 | exp Back Pain/ |
| 19 | Coccyx/in [Injuries] |
| 20 | Intervertebral Disc Degeneration/ |
| 21 | Intervertebral Disc Displacement/ |
| 22 | Lumbar Vertebrae/in [Injuries] |
| 23 | Lumbosacral Region/in [Injuries] |
| 24 | Osteoarthritis, Spine/ |
| 25 | Piriformis Muscle Syndrome/ |
| 26 | Radiculopathy/ |
| 27 | Sciatica/ |
| 28 | Spinal Diseases/ |
| 29 | Spinal Stenosis/ |
| 30 | Spondylosis/ |
| 31 | Spondylolysis/ |
| 32 | Spondylolisthesis/ |
| 33 | Thoracic Injuries/ |
| 34 | Thoracic Vertebrae/ |
| 35 | (back adj3 (ache* or injur* or pain*)).ab,ti. |
| 36 | (backache* adj3 (injur* or pain*)).ab,ti. |
| 37 | (back pain or back-pain).ab,ti. |
| 38 | (lumbar disc* adj3 (extruded or degenerat* or herniat* or prolapse* or sequestered or slipped)).ab,ti. |
| 39 | (lumbar disk* adj3 (extruded or degenerat* or herniat* or prolapse* or sequestered or slipped)).ab,ti. |
| 40 | "low* back pain".ab,ti. |
| 41 | (lumbar adj3 (pain or facet or nerve root* or osteoarthritis or radicul* or spinal stenosis or spondylo* or zygapophys*)).ab,ti. |
| 42 | "Piriformis syndrome*".ab,ti. |
| 43 | radiculopathy.ab,ti. |
| 44 | (sacral adj2 pain*).ab,ti. |
| 45 | ((spine or spinal) adj4 (condition* or disable* or disabilit* or disorder* or pain or stenos?s)).ab,ti. |
| 46 | spondylosis.ab,ti. |
| 47 | spondylolysis.ab,ti. |
| 48 | spondylolisthesis.ab,ti. |
| 49 | (thoracic adj4 (injur* or pain or spine or spinal)).ab,ti. |
| 50 | (T-spine or T-spinal).ab,ti. |
| 51 | or/17-50 [**back pain] |
| 52 | Acupressure/ |
| 53 | Acupuncture/ |
| 54 | exp Acupuncture Therapy/ |
| 55 | "Bedding and Linens"/ |
| 56 | Behavior Therapy/ |
| 57 | exp Biofeedback, Psychology/ |
| 58 | exp Cognitive Behavioral Therapy/ |
| 59 | Combined Modality Therapy/ |
| 60 | Community-Based Participatory Research/ |
| 61 | Community Health Services/ |
| 62 | Community Participation/ |
| 63 | Complementary Therapies/ |
| 64 | Cryotherapy/ |
| 65 | exp Diathermy/ |
| 66 | exp Electric Stimulation Therapy/ |
| 67 | Electroacupuncture/ |
| 68 | Ergonomics/ |
| 69 | exp Exercise/ |
| 70 | exp Exercise Movement Techniques/ |
| 71 | exp Exercise Therapy/ |
| 72 | Fluid Therapy/ |
| 73 | High-Energy Shock Waves/tu [Therapeutic Use] |
| 74 | Immobilization/ |
| 75 | Hot Temperature/tu [Therapeutic Use] |
| 76 | exp Hydrotherapy/ |
| 77 | Laser Therapy, Low-Level/ |
| 78 | Low-Level Light Therapy/ |
| 79 | Magnetic Field Therapy/ |
| 80 | Magnetics/tu [Therapeutic Use] |
| 81 | Massage/ |
| 82 | exp Medicine, Chinese Traditional/ |
| 83 | exp Musculoskeletal Manipulations/ |
| 84 | Patient Education as Topic/ |
| 85 | Physical Therapy Modalities/ |
| 86 | Self Care/ |
| 87 | Self-Help Devices/ |
| 88 | Physical Fitness/ |
| 89 | Restraint, Physical/ |
| 90 | Transcutaneous Electric Nerve Stimulation/ |
| 91 | Vibration/tu [Therapeutic Use] |
| 92 | Wheelchairs/ |
| 93 | acupressure.ab,ti. |
| 94 | "acupunctur*".ab,ti. |
| 95 | (advice or advise or advised).ab,ti. |
| 96 | alexander technique.ab,ti. |
| 97 | "assistive device*".ab,ti. |
| 98 | "back belt*".ab,ti. |
| 99 | "back school*".ab,ti. |
| 100 | (back adj2 work).ab,ti. |
| 101 | (braces or brace or bracing).ab,ti. |
| 102 | canes.ab,ti. |
| 103 | chiropract*.ab,ti. |
| 104 | "cognitive behavioral therap*".ab,ti. |
| 105 | "cognitive behavioural therap*".ab,ti. |
| 106 | (cold adj3 (therap* or pack* or compress or massage or immersion or soak or treatment or therap*)).ab,ti. |
| 107 | "core stabili*".ab,ti. |
| 108 | (corset or corsets).ab,ti. |
| 109 | crutches.ab,ti. |
| 110 | cryotherap*.ab,ti. |
| 111 | "deep tissue therap*".ab,ti. |
| 112 | diathermy.ab,ti. |
| 113 | (electric* adj3 (stimulation or EMS or heating pad*)).ab,ti. |
| 114 | electro-acupuncture.ab,ti. |
| 115 | (electrogalvanic stimulation or EGS).ab,ti. |
| 116 | (electromagnet* and (radiation or therap*)).ab,ti. |
| 117 | electromodalit*.ab,ti. |
| 118 | electrotherapy.ab,ti. |
| 119 | (exercise or exercises or exercising).ab,ti. |
| 120 | (flexion-distraction or flexion distraction).ab,ti. |
| 121 | fluidotherap*.ab,ti. |
| 122 | galvanic stimulation.ab,ti. |
| 123 | (H-Wave Device Stimulation or HWDS).ab,ti. |
| 124 | ((heat* or hot) adj3 (therap* or pack* or compress or massage or lamp or pad or bath or soak or tub or bottle or superficial or therapeutic)).ab,ti. |
| 125 | (high energy shock wave* or high-energy shock wave* or HESW).ab,ti. |
| 126 | "hydrotherap*".ab,ti. |
| 127 | (ice adj3 (therap* or pack* or compress or massage or immersion or soak or treatment or therap*)).ab,ti. |
| 128 | "interferential current*".ab,ti. |
| 129 | infrared.ab,ti. |
| 130 | iontophoresis.ab,ti. |
| 131 | electroanalgesia.ab,ti. |
| 132 | ergonomic*.ab,ti. |
| 133 | kinesiotap*.ab,ti. |
| 134 | (laser* adj3 (phototherapy or irradiation or biostimulation or light or therap*)).ab,ti. |
| 135 | "low level laser*".ab,ti. |
| 136 | "lumbar support*".ab,ti. |
| 137 | (magnetic adj3 (necklace* or therap* or bracelet*)).ab,ti. |
| 138 | (manipulat* adj3 (therap* or treatment* or spinal or osteopath*)).ab,ti. |
| 139 | "manual therap*".ab,ti. |
| 140 | Microcurrent Electrical Neuromuscular Stimulation.ab,ti. |
| 141 | microwave*.ab,ti. |
| 142 | ((moblisation or mobilization) adj4 (osteopath* or orthopedic* or orthopaedic* or lumbar or spinal)).ab,ti. |
| 143 | "moist air bath*".ab,ti. |
| 144 | moxibustion.ab,ti. |
| 145 | ((multimodal* or multi-modal* or multi modal*) adj4 (treatment* or approach or care or therap* or procedure* or package* or manage*)).ab,ti. |
| 146 | muscle activation.ab,ti. |
| 147 | "muscle energy technique*".ab,ti. |
| 148 | myofascial release.ab,ti. |
| 149 | (Neuromuscular Electrical Stimulation or NMES).ab,ti. |
| 150 | orthotic*.ab,ti. |
| 151 | "passive modalit*".ab,ti. |
| 152 | (patient* adj3 (educat* or train*)).ab,ti. |
| 153 | "Percutaneous Electric* Nerve Stimulation".ab,ti. |
| 154 | (physical adj therap*).ab,ti. |
| 155 | physiotherap*.ab,ti. |
| 156 | photo-acupuncture.ab,ti. |
| 157 | pillow*.ab,ti. |
| 158 | pilates.ab,ti. |
| 159 | (postur* adj3 (correct* or educat* or instruct* or train*)).ab,ti. |
| 160 | (pulsed adj3 (electromagnetic or magnetic or radio frequency or energy)).ab,ti. |
| 161 | radiant light.ab,ti. |
| 162 | Russian stimulation.ab,ti. |
| 163 | "seat adj cushion*".ab,ti. |
| 164 | (self-manage* or self manage*).ab,ti. |
| 165 | (short wave* or short-wave*).ab,ti. |
| 166 | ((shockwave* or shock wave* or shock-wave*) adj3 (ultrasonic or therap* or radiation)).ab,ti. |
| 167 | "soft tissue therap*".ab,ti. |
| 168 | "spray and stretch".ab,ti. |
| 169 | strain-counterstrain.ab,ti. |
| 170 | strengthen*.ab,ti. |
| 171 | stretching.ab,ti. |
| 172 | (tape or taping).ab,ti. |
| 173 | thoracolumbosacral orthosis.ab,ti. |
| 174 | traction.ab,ti. |
| 175 | traditional Chinese medicine.ab,ti. |
| 176 | (transcutaneous electrical stimulation or TENS).ab,ti. |
| 177 | ultrasound.ab,ti. |
| 178 | vapocoolant spray.ab,ti. |
| 179 | "vibration therap*".ab,ti. |
| 180 | walkers.ab,ti. |
| 181 | "walking adj3 aid*".ab,ti. |
| 182 | "warm compress*".ab,ti. |
| 183 | whirlpool*.ab,ti. |
| 184 | yoga.ab,ti. |
| 185 | or/52-184 [**interventions] |
| 186 | Case-Control Studies/ |
| 187 | Cohort Studies/ |
| 188 | Controlled Clinical Trials as Topic/ |
| 189 | Epidemiologic Studies/ |
| 190 | Epidemiology/ |
| 191 | Follow-Up Studies/ |
| 192 | Longitudinal Studies/ |
| 193 | Prospective Studies/ |
| 194 | Retrospective Studies/ |
| 195 | Randomized Controlled Trials as Topic/ |
| 196 | ((case control or case-control) adj3 (stud* or design*)).ab,ti. |
| 197 | (cohort adj3 (stud* or design* or analysis)).ab,ti. |
| 198 | controlled clinical trial.pt. |
| 199 | "epidemiolog*".ab,ti. |
| 200 | ((followup or follow-up) adj3 (stud* or design* or analysis)).ab,ti. |
| 201 | (longitudinal* adj3 (stud* or design* or analysis)).ab,ti. |
| 202 | (prospective adj3 (stud* or design* or analysis)).ab,ti. |
| 203 | (random* and (control* or clinical or allocat*)).ab,ti. |
| 204 | randomized controlled trial.pt. |
| 205 | (retrospective adj3 (stud* or design*)).ab,ti. |
| 206 | or/186-205 [**study designs_effectiveness] |
| 207 | 16 and 51 and 185 and 206 |
| 208 | Anthropology, Cultural/ |
| 209 | Attitude/ |
| 210 | Awareness/ |
| 211 | Behavioral Research/ |
| 212 | Diary as Topic/ |
| 213 | Emotions/ |
| 214 | Ethnology/ |
| 215 | Ethnopsychology/ |
| 216 | Focus Groups/ |
| 217 | Grounded Theory/ |
| 218 | Interview, Psychological/ |
| 219 | Interviews as Topic/ |
| 220 | Mindfulness/ |
| 221 | Motivation/ |
| 222 | Narration/ |
| 223 | Observation/ |
| 224 | Perception/ |
| 225 | Personal Narratives as Topic/ |
| 226 | Personal Satisfaction/ |
| 227 | Qualitative Research/ |
| 228 | Self Report/ |
| 229 | "Surveys and Questionnaires"/ |
| 230 | Tape Recording/ |
| 231 | Thinking/ |
| 232 | Video Recording/ or Videotape Recording/ |
| 233 | (attitude* or aware* or belief* or believe* or experience* or mindfulness or motivation or opinion* or perception* or perspective*).ab,ti. |
| 234 | ((audio adj record*) or audiorecord* or audiotap*).ab,ti. |
| 235 | ((behavioral or behavioural) adj2 research).ab,ti. |
| 236 | biographical method*.ab,ti. |
| 237 | (constant adj2 (comparative or comparison)).ab,ti. |
| 238 | ((content or conversation or discourse) adj2 analys*).ab,ti. |
| 239 | descriptive research.ab,ti. |
| 240 | (diary or diaries).ab,ti. |
| 241 | emotions.ab,ti. |
| 242 | ethnograph*.ab,ti. |
| 243 | ethnology.ab,ti. |
| 244 | ethnopsychology.ab,ti. |
| 245 | feelings.ab,ti. |
| 246 | (field adj2 (notes or research or study or studies)).ab,ti. |
| 247 | (focus adj2 group*).ab,ti. |
| 248 | framework analysis.ab,ti. |
| 249 | grounded theory.ab,ti. |
| 250 | interview*.ab,ti. |
| 251 | life world.ab,ti. |
| 252 | lived experience.ab,ti. |
| 253 | (meaning or meanings).ab,ti. |
| 254 | (narrative* or narration*).ab,ti. |
| 255 | (observe* or observation*).ab,ti. |
| 256 | (open adj ended).ab,ti. |
| 257 | phenomenology.ab,ti. |
| 258 | purposive sampl*.ab,ti. |
| 259 | qualitative.ab,ti. |
| 260 | questionnaire*.ab,ti. |
| 261 | (realist adj3 (review* or research or synthesis)).ab,ti. |
| 262 | satisfaction.ab,ti. |
| 263 | self report*.ab,ti. |
| 264 | semantic analysis.ab,ti. |
| 265 | standpoint*.ab,ti. |
| 266 | (story or stories).ab,ti. |
| 267 | survey*.ab,ti. |
| 268 | (theme* or thematic).ab,ti. |
| 269 | (theoretical adj2 (sampl* or saturation)).ab,ti. |
| 270 | (thoughts or thinking).ab,ti. |
| 271 | ((video adj record*) or videorecord* or videotap*).ab,ti. |
| 272 | or/208-271 [**experience/qualitative] |
| 273 | "Costs and Cost Analysis"/ |
| 274 | exp Cost-Benefit Analysis/ |
| 275 | Quality-Adjusted Life Years/ |
| 276 | Economics, Medical/ |
| 277 | (economic* adj4 (evaluat* or stud*)).ab,ti. |
| 278 | (health economic* adj4 (evaluat* or stud*)).ab,ti. |
| 279 | ((cost-utility or cost utility) adj4 (stud* or analys*)).ab,ti. |
| 280 | ((cost-benefit or cost benefit) adj4 (stud* or analys*)).ab,ti. |
| 281 | (CEA or CUA or CBA).ab,ti. |
| 282 | ((cost-effective* or cost effective*) adj4 (analys* or stud*)).ab,ti. |
| 283 | (economic* adj4 (impact or value or factor* or analys*)).ab,ti. |
| 284 | (cost* adj4 (health care or analys* or savings or hospital or medical or utilit* or effective* or efficac* or benefit* or consequence* or unit*)).ab,ti. |
| 285 | (decision adj1 (tree* or analy* or model*)).ab,ti. |
| 286 | economics.fs. |
| 287 | (qol or qoly or qolys or hrqol or qaly or qalys or qale or qales).ab,ti. |
| 288 | (sensitivity analys* or "willingness to pay" or quality-adjusted life year* or quality adjusted life year* or quality-adjusted life expectanc* or quality adjusted life expectanc*).ab,ti. |
| 289 | (markov* or monte carlo*).ab,ti. |
| 290 | or/273-289 [**cost effectiveness] |
| 291 | Delivery of Health Care/ |
| 292 | Delivery of Health Care, Integrated/ |
| 293 | Health Planning/ |
| 294 | Health Promotion/ |
| 295 | Health Services Administration/ |
| 296 | Integrative Medicine/ |
| 297 | Interprofessional Relations/ |
| 298 | Patient Care Management/ |
| 299 | (approach* adj3 (collaborative or complementary or comprehensive or innovative or integrated)).ab,ti. |
| 300 | barrier*.ab,ti. |
| 301 | facilitator*.ab,ti. |
| 302 | ((health care or healthcare or health-care) adj3 (clinic or clinics or delivery or implement* or intervention* or model* or plan* or process* or program*or services or strateg* or system* or team*)).ab,ti. |
| 303 | implement*.ab,ti. |
| 304 | (innovate* adj3 (intervention* or model* or plan* or process* or program*or strateg* or system*)).ab,ti. |
| 305 | (model* adj care).ab,ti. |
| 306 | ((integrated or interdisciplinary or interprofessional or multidisciplinary) adj3 (care or clinic or clinics or intervention* or model* or plan* or process* or program*or strateg* or system* or challenge* or benefit* or success* or constrain* or difficult* or enhanc* or influen* or interfer* or motivat* or obstruct* or problem* or promot* or restrain* or restrict* or disincentive* or factor* or capacity or enabler*)).ab,ti. |
| 307 | (pathway* adj3 (clinical or care)).ab,ti. |
| 308 | (program* adj3 (assess* or evaluat*)).ab,ti. |
| 309 | or/291-308 [**implementation] |
| 310 | 16 and 51 and 185 and (206 or 272 or 290 or 309) |
