# Supplementary material for: Rehabilitation of back pain in the pediatric population: a mixed studies systematic review
Source: Chiropr Man Therap. 2024 May 8;32:14. doi: 10.1186/s12998-024-00538-z (PMC11080233; doi:10.1186/s12998-024-00538-z)
Supplement: Supplementary file 3 — Additional file 3: Exclusion reasons for studies excluded in full text screening. [file 12998_2024_538_MOESM3_ESM.docx]

**Additional file 3. Exclusion reasons for studies excluded in full text screening**

***Ineligible research question (8 full text articles)***

1. Anderson K, Sarwark JF, Conway JJ, Logue ES, Schafer MF. Quantitative assessment with SPECT imaging of stress injuries of the pars interarticularis and response to bracing. Journal of pediatric orthopedics. 2000;20(1):28-33.

2. Cottalorda J, Bourelle S, Gautheron V. Effects of backpack carrying in children. Orthopedics. 2004;27(11):1172-5.

3. Kongsted A, Vach W, Axo M, Bech RN, Hestbaek L. Expectation of recovery from low back pain: a longitudinal cohort study investigating patient characteristics related to expectations and the association between expectations and 3-month outcome. Spine. 2014;39(1):81-90.

4. Louw Q, Kriel Rena I, Brink Y, van Niekerk S-M, Tawa N. Perspectives of spinal health within the school setting in a South African rural region: A qualitative study. Work (Reading, Mass). 2021;69(1):141-55.

5. Mierau DR, Cassidy JD, Hamin T, Milne RA. Sacroiliac joint dysfunction and low back pain in school aged children. J Manipulative Physiol Ther. 1984;7(2):81-4.

6. Newcomer K, Sinaki M, Wollan PC. Physical activity and four-year development of back strength in children. American Journal of Physical Medicine and Rehabilitation. 1997;76(1):52-8.

7. Noblet T, Marriott J, Hensman-Crook A, O'Shea S, Friel S, Rushton A. Independent prescribing by advanced physiotherapists for patients with low back pain in primary care: A feasibility trial with an embedded qualitative component. PloS one. 2020;15(3):e0229792.

8. Pereira MG, Roios E, Pereira M. Functional disability in patients with low back pain: the mediator role of suffering and beliefs about pain control in patients receiving physical and chiropractic treatment. Brazilian Journal of Physical Therapy / Revista Brasileira de Fisioterapia. 2017;21(6):465-72.

***Ineligible population (235 full text articles)***

1. Promoting healthy backs in schools: A evaluative study. Health Education. 2007;107(5):463-79.

2. An ergonomics training program for student notebook computer users: Preliminary outcomes of a six-year cohort study. Work: Journal of Prevention, Assessment & Rehabilitation. 2013;44(2):221-30.

3. Adeyemi AJ, Lasisi OI, Ojile P, Abdulkadir M. The effect of furniture intervention on the occurrence of musculoskeletal disorders and academic performance of students in North-West Nigeria. Work (Reading, Mass). 2020;65(1):195-203.

4. Akca Nazan K, Aydin G, Gumus K. Effect of Body Mechanics Brief Education in the Clinical Setting on Pain Patients with Lumbar Disc Hernia: A Randomized Controlled Trial. International Journal of Caring Sciences. 2017;10(3):1498-506.

5. AlBedah A, Khalil M, Elolemy A, Hussein Asim A, AlQaed M, Al M, et al. The Use of Wet Cupping for Persistent Nonspecific Low Back Pain: Randomized Controlled Clinical Trial. Journal of Alternative & Complementary Medicine. 2015;21(8):504-8.

6. Albert HB, Manniche C. The efficacy of systematic active conservative treatment for patients with severe sciatica: a single-blind, randomized, clinical, controlled trial. Spine. 2012;37(7):531-42.

7. Alricsson M, Werner S. The effect of pre-season dance training on physical indices and back pain in elite cross-country skiers: a prospective controlled intervention study. British Journal of Sports Medicine. 2004;38(2):148-53.

8. Alzahrani H, Cheng SWM, Shirley D, Mackey M, Stamatakis E. Physical Activity and Health-Related Quality of Life in People With Back Pain: A Population-Based Pooled Study of 27,273 Adults. Journal of physical activity & health. 2019:1-12.

9. Andersen L. Lumbar spondylolysis treated with cerclage using the method of the 80's. [Danish] Lumbal arkolyse behandlet med ottetalscerclage. Ugeskrift for laeger. 1994;156(26):3888-90.

10. Anderson K, Sarwark JF, Conway JJ, Logue ES, Schafer MF. Quantitative assessment with SPECT imaging of stress injuries of the pars interarticularis and response to bracing. Journal of Pediatric Orthopedics. 2000;20(1):28-33.

11. Annen M, Peterson C, Humphreys BK. Comparison of Treatment Outcomes in Nonspecific Low-Back Pain Patients With and Without Modic Changes Who Receive Chiropractic Treatment. Journal of Manipulative & Physiological Therapeutics. 2018;41(7):561-70.

12. Apeldoorn AT, Ostelo RW, van H, Fritz JM, de V, H C, et al. The cost-effectiveness of a treatment-based classification system for low back pain: design of a randomised controlled trial and economic evaluation. BMC Musculoskeletal Disorders. 2010;11:58.

13. Aspegren D, Enebo BA, Miller M, White L, Akuthota V, Hyde TE, et al. Functional scores and subjective responses of injured workers with back or neck pain treated with chiropractic care in an integrative program: a retrospective analysis of 100 cases. Journal of Manipulative & Physiological Therapeutics. 2009;32(9):765-71.

14. Atlas SJ, Deyo RA, Keller RB, Chapin AM, Patrick DL, Long JM, et al. The Maine Lumbar Spine Study, Part II. 1-year outcomes of surgical and nonsurgical management of sciatica. Spine. 1996;21(15):1777-86.

15. Avrahami D, Potvin Jim R. The clinical and biomechanical effects of fascial-muscular lengthening therapy on tight hip flexor patients with and without low back pain. Journal of the Canadian Chiropractic Association. 2014;58(4):444-55.

16. Badke MB, Boissonnault WG. Changes in disability following physical therapy intervention for patients with low back pain: dependence on symptom duration. Archives of Physical Medicine & Rehabilitation. 2006;87(6):749-56.

17. Bamford A, Nation A, Durrell S, Andronis L, Rule E, McLeod H. Implementing the Keele stratified care model for patients with low back pain: An observational impact study. BMC Musculoskeletal Disorders. 2017;18 (1) (no pagination)(66).

18. Banbury P, Feenan K, Allcock N. Experiences of analgesic use in patients with low back pain. British journal of nursing (Mark Allen Publishing). 2008;17(19):1215-8.

19. Bao S, Silverstein B, Stewart K. Evaluation of an ergonomics intervention among Nicaraguan coffee harvesting workers. Ergonomics. 2013;56(2):166-81.

20. Barbosa AC, Martins FL, Barbosa MC, Dos S, R T. Manipulation and selective exercises decrease pelvic anteversion and low-back pain: a pilot study. Journal of Back & Musculoskeletal Rehabilitation. 2013;26(1):33-6.

21. Bendix AF, Bendix T, Lund C, Kirkbak S, Ostenfeld S. Comparison of three intensive programs for chronic low back pain patients: a prospective, randomized, observer-blinded study with one-year follow-up. Scandinavian Journal of Rehabilitation Medicine. 1997;29(2):81-9.

22. Beneciuk JM, Robinson ME, George SZ. Low back pain subgroups using fear-avoidance model measures: results of a cluster analysis. Clinical Journal of Pain. 2012;28(8):658-66.

23. Bhushan V, Sahay P, Alam S, Ranjana, Equebal A. Efficacy of Maitland Mobilization and Lumbar Segmental Stabilization Exercises as Compared to Lumbar Segmental Stabilization Exercises in Subjects with Mechanical Low Back Pain: A Randomized Controlled Trial. Indian Journal of Physiotherapy & Occupational Therapy. 2016;10(4):113-8.

24. Blanda J, Bethem D, Moats W, Lew M. Defects of pars interarticularis in athletes: a protocol for nonoperative treatment. Journal of Spinal Disorders. 1993;6(5):406-11.

25. Broadhurst NA. Pelvic dysfunction. Journal of Neurological and Orthopaedic Medicine and Surgery. 1994;15(3):127-9.

26. Brodin H. Inhibition-facilitation technique for lumbar pain treatment. Acta Belgica - Medica Physica. 1983;6(1):31-5.

27. Bronfort G. [Chiropractic treatment of low backache. A prospective study]. Ugeskrift for Laeger. 1985;147(20):1611-8.

28. Brooks C, Siegler JC, Cheema BS, Marshall PW. No relationship between body mass index and changes in pain and disability after exercise rehabilitation for patients with mild to moderate chronic low back pain. Spine. 2013;38(25):2190-5.

29. Brotz D, Kuker W, Maschke E, Wick W, Dichgans J, Weller M. A prospective trial of mechanical physiotherapy for lumbar disk prolapse. Journal of Neurology. 2003;250(6):746-9.

30. Burton AK, Tillotson KM, Cleary J. Single-blind randomised controlled trial of chemonucleolysis and manipulation in the treatment of symptomatic lumbar disc herniation. European Spine Journal. 2000;9(3):202-7.

31. Burton AK, Waddell G, Tillotson KM, Summerton N. Information and advice to patients with back pain can have a positive effect: A randomized controlled trial of a novel educational booklet in primary care. Spine. 1999;24(23):2484-91.

32. Buttagat V, Eungpinichpong W, Chatchawan U, Arayawichanon P. Therapeutic effects of traditional Thai massage on pain, muscle tension and anxiety in patients with scapulocostal syndrome: a randomized single-blinded pilot study. Journal of Bodywork & Movement Therapies. 2012;16(1):57-63.

33. Buttagat V, Narktro T, Onsrira K, Pobsamai C. Short-term effects of traditional Thai massage on electromyogram, muscle tension and pain among patients with upper back pain associated with myofascial trigger points. Complementary Therapies in Medicine. 2016;28:8-12.

34. Candy E, Farewell DJ-HCSLWRAS. Effect of a high-density foam seating wedge on back pain intensity when used by 14 to 16-year-old school students: a randomised controlled trial. 2012;98:301.

35. Candy E, Shepstone L, Watts R. Does the introduction of a simple wedge to school seating reduce adolescent back pain?...including commentary by Limon S. International Journal of Therapy & Rehabilitation. 2004;11(10):462-6.

36. Candy EA, Farewell D, Jerosch-Herold C, Shepstone L, Watts RA, Stephenson RC. Effect of a high-density foam seating wedge on back pain intensity when used by 14 to 16-year-old school students: A randomised controlled trial. Physiotherapy (United Kingdom). 2012;98(4):301-7.

37. Cao Y, Wang Y. [Acute lumbar sprain treated with massage combined with acupuncture at different distal acupoints: a randomized controlled trial]. Zhongguo Zhenjiu. 2015;35(5):453-7.

38. Casserley-Feeney SN, Daly L, Hurley DA. The access randomized clinical trial of public versus private physiotherapy for low back pain. Spine. 2012;37(2):85-96.

39. Cheing GL, Hui-Chan CW. Transcutaneous electrical nerve stimulation: nonparallel antinociceptive effects on chronic clinical pain and acute experimental pain. Archives of Physical Medicine & Rehabilitation. 1999;80(3):305-12.

40. Chen M, Chen R, Xiong J, Chi Z, Sun J, Su T, et al. Evaluation of different moxibustion doses for lumbar disc herniation: multicentre randomised controlled trial of heat-sensitive moxibustion therapy. Acupuncture in medicine : journal of the British Medical Acupuncture Society. 2012;30(4):266-72.

41. Cherkin DC, MacCornack FA. Patient evaluations of low back pain care from family physicians and chiropractors. Western Journal of Medicine. 1989;150(3):351-5.

42. Chiradejnant A, Maher CG, Latimer J, Stepkovitch N. Efficacy of "therapist-selected" versus "randomly selected" mobilisation techniques for the treatment of low back pain: a randomised controlled trial. Australian Journal of Physiotherapy. 2003;49(4):233-41.

43. Chuang LH, Soares MO, Tilbrook H, Cox H, Hewitt CE, Aplin J, et al. A pragmatic multicentered randomized controlled trial of yoga for chronic low back pain: economic evaluation. Spine. 2012;37(18):1593-601.

44. Coan RM, Wong G, Ku SL, Chan YC, Wang L, Ozer FT, et al. The acupuncture treatment of low back pain: a randomized controlled study. American Journal of Chinese Medicine. 1980;8(1-2):181-9.

45. Cooper K, Smith BH, Hancock E. Patients' perceptions of self-management of chronic low back pain: evidence for enhancing patient education and support. Physiotherapy. 2009;95(1):43-50.

46. Czaprowski D, Leszczewska J, Kolwicz A, Pawlowska P, Afeltowicz-Mich A, Sitarski D, et al. Comparison of influence of three physiotherapeutic techniques: postisometric relaxation, kinetic control and stabilization exercises on the flexibility of pelvic girdle muscles: prospective, randomized, single blinded study. Studies in Health Technology & Informatics. 2012;176:499-.

47. Czarnecki ML, Garwood MM, Weisman SJ. Advanced practice nurse-directed telephone management of acute pain following pediatric spinal fusion surgery. Journal for Specialists in Pediatric Nursing. 2007;12(3):159-69.

48. Daltroy LH, Iversen MD, Larson MG, Lew R, Wright E, Ryan J, et al. A controlled trial of an educational program to prevent low back injuries. New England Journal of Medicine. 1997;337(5):322-8.

49. Darlow B, Dean S, Perry M, Mathieson F, Baxter GD, Dowell A. Easy to Harm, Hard to Heal: Patient Views About the Back. Spine. 2015;40(11):842-50.

50. Darlow B, Dowell A, Baxter GD, Mathieson F, Perry M, Dean S. The enduring impact of what clinicians say to people with low back pain. Annals of family medicine. 2013;11(6):527-34.

51. de B, Dawid, Bester Charmaine M. The effect of lumbosacral manipulation on growing pains. Health SA Gesondheid. 2015;20(1):75-82.

52. Dechow E, Davies RK, Carr AJ, Thompson PW. A randomized, double-blind, placebo-controlled trial of sclerosing injections in patients with chronic low back pain. Rheumatology. 1999;38(12):1255-9.

53. Dehlin O, Berg S, Hedenrud B, Andersson G, Grimby G. Muscle training, psychological perception of work and low-back symptoms in nursing aides. The effect of trunk and quadriceps muscle training on the psychological perception of work and on the subjective assessment of low-back insufficiency. A study in a geriatric hospital. Scandinavian Journal of Rehabilitation Medicine. 1978;10(4):201-9.

54. Delitto A, Cibulka MT, Erhard RE, Bowling RW, Tenhula JA. Evidence for use of an extension-mobilization category in acute low back syndrome: a prescriptive validation pilot study. Physical Therapy. 1993;73(4):216-22; discussion 23.

55. Desmoulin GT, Yasin NI, Chen DW. Initial results using KHAN KINETIC TREATMENT as a low back pain treatment option. Journal of Musculoskeletal Pain. 2007;15(3):91-102.

56. Dey P, Simpson CW, Collins SI, Hodgson G, Dowrick CF, Simison AJ, et al. Implementation of RCGP guidelines for acute low back pain: a cluster randomised controlled trial. British Journal of General Practice. 2004;54(498):33-7.

57. Dineshkumar Sharma K, Dibyendunarayan B, Ramalingam A, Thangamani. EFFECT OF ABDOMINAL DRAWING-IN MANEUVER ALONG WITH RESISTED ANKLE DORSI-FLEXION TO ACTIVATE TRANSVERSE ABDOMINIS MUSCLE IN CHRONIC NON-SPECIFIC LOW BACK PAIN. Romanian Journal of Physical Therapy / Revista Romana de Kinetoterapie. 2015;21(36):40-7.

58. Dissing KB, Hartvigsen J, Wedderkopp N, Hestbaek L. Conservative care with or without manipulative therapy in the management of back and/or neck pain in Danish children aged 9-15: a randomised controlled trial nested in a school-based cohort. BMJ open. 2018;8(9):e021358.

59. Dissing KB, Vach W, Hartvigsen J, Wedderkopp N, Hestbaek L. Potential treatment effect modifiers for manipulative therapy for children complaining of spinal pain.Secondary analyses of a randomised controlled trial. Chiropractic and Manual Therapies. 2019;27 (1) (no pagination)(59).

60. Drks. Cluster-randomized, controlled evaluation of a teacher led multi factorial school based back education program for 10 to 12-year old children. 2018.

61. Dsa Cassandra F, Rengaramanujam K, Kudchadkar Mahendra S. To assess the effect of Modified Pilates Compared to Conventional Core Stabilization Exercises on Pain and Disability in Chronic Non-Specific Low Back Pain-Randomized Controlled Trial. Indian Journal of Physiotherapy & Occupational Therapy. 2014;8(3):202-7.

62. Du HG, Wei H, Jiang Z, Wang HM, Ye SL, Song HQ, et al. [Case-control study on three spinal rotation manipulations for the treatment of lumbar disc herniation]. Zhongguo Gushang. 2016;29(5):444-8.

63. Dullien S, Grifka J, Jansen P. Cluster-randomized, controlled evaluation of a teacher led multi factorial school based back education program for 10 to 12-year old children. BMC Pediatrics. 2018;18 (1) (no pagination)(312).

64. El R, Takemitsu M, Glutting J, Shah SA. Effect of sports modification on clinical outcome in children and adolescent athletes with symptomatic lumbar spondylolysis. American journal of physical medicine & rehabilitation / Association of Academic Physiatrists. 2013;92(12):1070-4.

65. Erhard RE, Delitto A, Cibulka MT. Relative effectiveness of an extension program and a combined program of manipulation and flexion and extension exercises in patients with acute low back syndrome. Physical Therapy. 1994;74(12):1093-100.

66. Evans C, Gilbert JR, Taylor W, Hildebrand A. A randomized controlled trial of flexion exercises, education, and bed rest for patients with acute low back pain. Physiotherapy Canada. 1987;39(2):96-101.

67. Faas A, Chavannes AW, van E, J T, Gubbels JW. A randomized, placebo-controlled trial of exercise therapy in patients with acute low back pain. Spine. 1993;18(11):1388-95.

68. Faas A, Chavannes AW, Van E, J TM, Gubbels JW. A randomized, placebo-controlled trial of exercise therapy in patients with acute low back pain. Spine. 1993;18(11):1388-95.

69. Ferreira PH, Ferreira ML, Maher CG, Refshauge K, Herbert RD, Hodges PW. Changes in recruitment of transversus abdominis correlate with disability in people with chronic low back pain. British journal of sports medicine. 2010;44(16):1166-72.

70. Fewster KM, Gallagher KM, Callaghan JP. The effect of standing interventions on acute low-back postures and muscle activation patterns. Applied Ergonomics. 2017;58:281-6.

71. Flynn T, Fritz J, Whitman J, Wainner R, Magel J, Rendeiro D, et al. A clinical prediction rule for classifying patients with low back pain who demonstrate short-term improvement with spinal manipulation. Spine. 2002;27(24):2835-43.

72. Ford JJ, Hahne AJ, Surkitt LD, Chan AY, Richards MC, Slater SL, et al. Individualised physiotherapy as an adjunct to guideline-based advice for low back disorders in primary care: a randomised controlled trial. British journal of sports medicine. 2016;50(4):237-45.

73. Fordyce WE, Brockway JA, Bergman JA, Spengler D. Acute back pain: a control-group comparison of behavioral vs traditional management methods. Journal of Behavioral Medicine. 1986;9(2):127-40.

74. Franche RL, Severin CN, Hogg-Johnson S, Cote P, Vidmar M, Lee H. The impact of early workplace-based return-to-work strategies on work absence duration: a 6-month longitudinal study following an occupational musculoskeletal injury. Journal of Occupational & Environmental Medicine. 2007;49(9):960-74.

75. Freburger JK, Carey TS, Holmes GM. Effectiveness of physical therapy for the management of chronic spine disorders: a propensity score approach. Physical Therapy. 2006;86(3):381-94.

76. Frost H, Klaber M, J A, Moser JS, Fairbank JC. Randomised controlled trial for evaluation of fitness programme for patients with chronic low back pain. BMJ. 1995;310(6973):151-4.

77. Frost H, Lamb SE, Klaber M, J A, Fairbank JC, Moser JS. A fitness programme for patients with chronic low back pain: 2-year follow-up of a randomised controlled trial. Pain. 1998;75(2-3):273-9.

78. Gaskell L, Enright S, Tyson S. The effects of a back rehabilitation programme for patients with chronic low back pain. Journal of Evaluation in Clinical Practice. 2007;13(5):795-800.

79. Gavin TM, Boscardin JB, Patwardhan AG, Bunch WH, Zindrick MR, Lorenz MA, et al. Preliminary results of orthotic treatment for chronic low back pain. Journal of Prosthetics & Orthotics (JPO). 1993;5(1):25-9.

80. Geldhof E, Cardon GDBIDCD. Back posture education in elementary schoolchildren: a 2-year follow-up study. 2007;16:841.

81. George SZ, Hirsh AT. Distinguishing patient satisfaction with treatment delivery from treatment effect: a preliminary investigation of patient satisfaction with symptoms after physical therapy treatment of low back pain. Archives of Physical Medicine & Rehabilitation. 2005;86(7):1338-44.

82. George SZ, Wittmer VT, Fillingim RB, Robinson ME. Comparison of graded exercise and graded exposure clinical outcomes for patients with chronic low back pain. Journal of Orthopaedic and Sports Physical Therapy. 2010;40(11):694-704.

83. Giles LG, Muller R. Chronic spinal pain syndromes: a clinical pilot trial comparing acupuncture, a nonsteroidal anti-inflammatory drug, and spinal manipulation. Journal of Manipulative & Physiological Therapeutics. 1999;22(6):376-81.

84. Glover JR, Morris JG, Khosla T. Back pain: a randomized clinical trial of rotational manipulation of the trunk. British journal of industrial medicine. 1974;31(1):59-64.

85. Gonzalez-Galvez N, Marcos-Pardo Pablo J, Albaladejo-Saura M, Lopez-Vivancos A, Vaquero-Cristobal R. Effects of a Pilates programme in spinal curvatures and hamstring extensibility in adolescents with thoracic hyperkyphosis: a randomised controlled trial. Postgraduate medical journal. 2022.

86. Grieves B, Menke JM, Pursel KJ. Cost minimization analysis of low back pain claims data for chiropractic vs medicine in a managed care organization. Journal of Manipulative & Physiological Therapeutics. 2009;32(9):734-9.

87. Guillory SA, Wilks SE, Isaza J. Perceived low back pain among consistent exercise participants: examining the effects of age and exertion. The Journal of the Louisiana State Medical Society : official organ of the Louisiana State Medical Society. 2008;160(1):27-34.

88. Haas M. Evaluation of physiotherapy using cost-utility analysis. Australian Journal of Physiotherapy. 1993;39(3):211-6.

89. Haase I, Haase K, Kladny B. [Evaluation of Inpatient Conservative Management of Acute, Subacute, and Chronic Back Pain]. Zeitschrift fur Orthopadie & Unfallchirurgie. 2018;156(2):184-92.

90. Hackett GI, Seddon D, Kaminski D. Electroacupuncture compared with paracetamol for acute low back pain. Practitioner. 1988;232(1443):163-4.

91. Hagen EM, Odelien KH, Lie SA, Eriksen HR. Adding a physical exercise programme to brief intervention for low back pain patients did not increase return to work. Scandinavian journal of public health. 2010;38(7):731-8.

92. Hamre HJ, Witt CM, Glockmann A, Troger W, Willich SN, Kiene H. Use and safety of anthroposophic medications in chronic disease: A 2-year prospective analysis. Drug Safety. 2006;29(12):1173-89.

93. Hamre HJ, Witt CM, Glockmann A, Wegscheider K, Ziegler R, Willich SN, et al. Anthroposophic vs. conventional therapy for chronic low back pain: A prospective comparative study. European Journal of Medical Research. 2007;12(7):302-10.

94. Harroud A, Labelle H, Joncas J, Mac-Thiong JM. Global sagittal alignment and health-related quality of life in lumbosacral spondylolisthesis. European Spine Journal. 2013;22(4):849-56.

95. Harts CC, Helmhout PH, de B, R A, Staal JB. A high-intensity lumbar extensor strengthening program is little better than a low-intensity program or a waiting list control group for chronic low back pain: A randomised clinical trial. Australian Journal of Physiotherapy. 2008;54(1):23-31.

96. He C, Chen P, Wang X, Ding M, Lan Q, Han M. The clinical effect of herbal magnetic corsets on lumbar disc herniation. Clinical Rehabilitation. 2006;20(12):1058-65.

97. Hechler T, Dobe M, Damschen U, Blankenburg M, Schroeder S, Kosfelder J, et al. The Pain Provocation Technique for Adolescents with Chronic Pain: Preliminary Evidence for Its Effectiveness. Pain Medicine. 2010;11(6):897-910.

98. Helmhout PH, Witjes M, Nijhuis VANDERSRW, Bron C, van A, Staal JB. The effects of lumbar extensor strength on disability and mobility in patients with persistent low back pain. The Journal of sports medicine and physical fitness. 2017;57(4):411-7.

99. Hemmila HM, Keinanen-Kiukaanniemi SM, Levoska S, Puska P. Does folk medicine work? A randomized clinical trial on patients with prolonged back pain. Archives of Physical Medicine and Rehabilitation. 1997;78(6):571-7.

100. Henchoz Y, Pinget C, Wasserfallen JB, Paillex R, de G, Norberg M, et al. Cost-utility analysis of a three-month exercise programme vs usual care following multidisciplinary rehabilitation for chronic low back pain. Journal of rehabilitation medicine : official journal of the UEMS European Board of Physical and Rehabilitation Medicine. 2010;42(9):846-52.

101. Hertzman-Miller RP, Morgenstern H, Hurwitz EL, Yu F, Adams AH, Harber P, et al. Comparing the satisfaction of low back pain patients randomized to receive medical or chiropractic care: results from the UCLA low-back pain study. American Journal of Public Health. 2002;92(10):1628-33.

102. Heyman E, Dekel H. Ergonomics for children: an educational program for elementary school. Work. 2009;32(3):261-5.

103. Hill JJ, Keating JL. Daily exercises and education for preventing low back pain in children: cluster randomized controlled trial. Physical therapy. 2015;95(4):507-16.

104. Hill Julia J, Keating Jennifer L. Research Report. Daily Exercises and Education for Preventing Low Back Pain in Children: Cluster Randomized Controlled Trial. 2015;95:507.

105. Hohmann CD, Stange R, Steckhan N, Robens S, Ostermann T, Paetow A, et al. The Effectiveness of Leech Therapy in Chronic Low Back Pain. Deutsches Arzteblatt International. 2018;115(47):785-92.

106. Hsieh LL, Kuo CH, Lee LH, Yen AM, Chien KL, Chen TH. Treatment of low back pain by acupressure and physical therapy: randomised controlled trial. BMJ. 2006;332(7543):696-700.

107. Hsieh LL, Kuo CH, Yen MF, Chen TH. A randomized controlled clinical trial for low back pain treated by acupressure and physical therapy. Preventive Medicine. 2004;39(1):168-76.

108. Hsieh LLC, Kuo CH, Yen MF, Chen THH. A randomized controlled clinical trial for low back pain treated by acupressure and physical therapy. Preventive Medicine. 2004;39(1):168-76.

109. Hsieh RL, Lee WC. One-shot percutaneous electrical nerve stimulation vs. transcutaneous electrical nerve stimulation for low back pain: Comparison of therapeutic effects. American Journal of Physical Medicine and Rehabilitation. 2002;81(11):838-43.

110. Huang GF, Zhang HXZTFYF. Time-dependent analgesic effect of electroacupuncture at Jiaji acupoint in patients with lumbar disc herniation and its intervention on related factors of plasma. 2006;10:1.

111. Iles RA, Taylor NF, Davidson M, O'Halloran P. An effective coaching intervention for people with low recovery expectations and low back pain: a content analysis. Journal of Back & Musculoskeletal Rehabilitation. 2014;27(1):93-101.

112. Iwamoto J, Takeda T, Wakano K. Returning athletes with severe low back pain and spondylolysis to original sporting activities with conservative treatment. Scandinavian Journal of Medicine and Science in Sports. 2004;14(6):346-51.

113. Jarzem PF, Harvey EJ, Arcaro N, Kaczorowski J. Transcutaneous electrical nerve stimulation [TENS] for chronic low back pain. Journal of Musculoskeletal Pain. 2005;13(2):3-9.

114. Johannsen F, Remvig L, Kryger P, Beck P, Warming S, Lybeck K, et al. Exercises for chronic low back pain: a clinical trial. Journal of Orthopaedic & Sports Physical Therapy. 1995;22(2):52-9.

115. Jose D-A, María, Kovacs Francisco M, Royuela A, Fernández-Serrano M, Gutiérrez-Fernández L, et al. Effectiveness of the Godelieve Denys-Struyf (GDS) Method in People With Low Back Pain: Cluster Randomized Controlled Trial. Physical Therapy. 2015;95(3):319-36.

116. Kempert H, Benore E, Heines R. Physical and occupational therapy outcomes: Adolescents' change in functional abilities using objective measures and self-report. Scandinavian Journal of Pain. 2017;14:60-6.

117. Kennedy S, Baxter GD, Kerr DP, Bradbury I, Park J, McDonough SM. Acupuncture for acute non-specific low back pain: a pilot randomised non-penetrating sham controlled trial. Complementary Therapies in Medicine. 2008;16(3):139-46.

118. Kettenmann B, Wille C, Lurie-Luke E, Walter D, Kobal G. Impact of continuous low level heatwrap therapy in acute low back pain patients: subjective and objective measurements. Clinical Journal of Pain. 2007;23(8):663-8.

119. Kimming A. Examination and treatment of 17-year-old students with pain in the back, hips, and knees. Pediatric Physical Therapy. 1997;9(1):2-11.

120. Kittang G, Melvaer T, Baerheim A. [Acupuncture contra antiphlogistics in acute lumbago]. Tidsskrift for Den Norske Laegeforening. 2001;121(10):1207-10.

121. Klaber M, J A, Carr J, Howarth E. High fear-avoiders of physical activity benefit from an exercise program for patients with back pain. Spine. 2004;29(11):1167-72; discussion 73.

122. Klaber M, J A, Chase SM, Portek I, Ennis JR. A controlled, prospective study to evaluate the effectiveness of a back school in the relief of chronic low back pain. Spine. 1986;11(2):120-2.

123. Kondziella W. Radiographic clinical and functional diagnosis and treatment of low back pain associated with pelvic malposition. [German] Beitrag zur klinischen und rontgenfunktionsanalytischen Diagnostik und Behandlung von Kreuzschmerzen bei Beckenverwringung. Schmerz. 1996;10(4):204-10.

124. Kongsted A, Vach W, Axo M, Bech RN, Hestbaek L. Expectation of recovery from low back pain: a longitudinal cohort study investigating patient characteristics related to expectations and the association between expectations and 3-month outcome. Spine. 2014;39(1):81-90.

125. Lane Tyler J, Lilley R, Hogg-Johnson S, LaMontagne Anthony D, Sim Malcolm R, Smith Peter M. A Prospective Cohort Study of the Impact of Return-to-Work Coordinators in Getting Injured Workers Back on the Job. Journal of Occupational Rehabilitation. 2018;28(2):298-306.

126. Lariviere C, Gagnon DH, Henry SM, Preuss R, Dumas JP. The Effects of an 8-Week Stabilization Exercise Program on Lumbar Multifidus Muscle Thickness and Activation as Measured With Ultrasound Imaging in Patients With Low Back Pain: An Exploratory Study. Pm & R. 2018;10(5):483-93.

127. Law SW, Szeto GPY, Chau WW, Carol C, Kwok Anthony WL, Lai HS, et al. Multi-disciplinary Orthopaedics Rehabilitation Empowerment (MORE) program: A new standard of care for injured workers in Hong Kong. Journal of Back & Musculoskeletal Rehabilitation. 2016;29(3):503-13.

128. Leeuw M, Goossens ME, van B, G J, de J, J R, et al. Exposure in vivo versus operant graded activity in chronic low back pain patients: results of a randomized controlled trial. Pain. 2008;138(1):192-207.

129. Lei LM, Huang JJ, Lin GQ, Chen JS, Pang YH, He YF, et al. [Lumbar intervertebral disc protrusion treated with Santong tuina therapy: a multi-central randomized controlled trial]. Zhongguo Zhenjiu. 2011;31(3):253-7.

130. Lewith GT, Turner GM. Retrospective analysis of the management of acute low back pain. Practitioner. 1982;226(1371):1614-8.

131. Liu JM, Tian WH, Tian JG, Li HT, Qi FJ, Fan Y, et al. [Observation on therapeutic effect of round-sharp needle of new nine-needle and elongated needle for piriformis syndrome with triple puncture method]. [Chinese]. Zhongguo zhen jiu = Chinese acupuncture & moxibustion. 2013;33(5):422-5.

132. Lofvander MB. Cognitive-behavioural treatment of chronic pain in primary care: A three-year follow-up. European Journal of General Practice. 2002;8(4):151-8.

133. Loisel P, Abenhaim L, Durand P, Esdaile JM, Suissa S, Gosselin L, et al. A population-based, randomized clinical trial on back pain management. Spine. 1997;22(24):2911-8.

134. Lopes S, Correia C, Felix G, Lopes M, Cruz A, Ribeiro F. Immediate effects of Pilates based therapeutic exercise on postural control of young individuals with non-specific low back pain: A randomized controlled trial. Complementary Therapies in Medicine. 2017;34:104-10.

135. Ma S, Ma J, Pan JN, Zhang XS. [Comparative research of lumbar disc herniation treated with acupuncture and snake moxibustion]. Zhongguo Zhenjiu. 2010;30(7):563-6.

136. Macedo CSG, Debiagi PC, Andrade FM. The Isostretching effect in the muscle strength of gluteus maximus, abdominal and the trunk extensor, incapacity and pain in patients with low back pain. Fisioterapia em Movimento. 2010;23(1):113-20.

137. Macedo LG, Latimer J, Maher CG, Hodges PW, McAuley JH, Nicholas MK, et al. Effect of motor control exercises versus graded activity in patients with chronic nonspecific low back pain: a randomized controlled trial. Physical therapy. 2012;92(3):363-77.

138. Majeed AS, Ts A, Sugunan A, Ms A. The effectiveness of a simplified core stabilization program (TRICCS-Trivandrum Community-based Core Stabilisation) for community-based intervention in chronic non-specific low back pain. Journal of orthopaedic surgery and research. 2019;14(1):86.

139. Malfliet A, Kregel J, Meeus M, Roussel N, Danneels L, Cagnie B, et al. Blended-Learning Pain Neuroscience Education for People With Chronic Spinal Pain: Randomized Controlled Multicenter Trial. Physical Therapy. 2018;98(5):357-68.

140. Manansala C, Passmore S, Pohlman K, Toth A, Olin G. Change in young people's spine pain following chiropractic care at a publicly funded healthcare facility in Canada. Complementary therapies in clinical practice. 2019;35:301-7.

141. Marchand S, Charest J, Li J, Chenard JR, Lavignolle B, Laurencelle L. Is TENS purely a placebo effect? A controlled study on chronic low back pain. Pain. 1993;54(1):99-106.

142. Mathews JA, Mills SB, Jenkins VM, Grimes SM, Morkel MJ, Mathews W, et al. Back pain and sciatica: controlled trials of manipulation, traction, sclerosant and epidural injections. British Journal of Rheumatology. 1987;26(6):416-23.

143. Mathews W, Morkel M, Mathews J. Manipulation and traction for lumbago and sciatica: physiotherapeutic techniques used in two controlled trials. Physiotherapy Practice. 1988;4(4):201-6.

144. Mattila VM, Sillanpaa P, Salo T, Laine HJ, Maenpaa H, Pihlajamaki H. Orthotic insoles do not prevent physical stress-induced low back pain. European Spine Journal. 2011;20(1):100-4.

145. Mierau D, Cassidy JD, McGregor M, Kirkaldy-Willis WH. A comparison of the effectiveness of spinal manipulative therapy for low back pain patients with and without spondylolisthesis. Journal of manipulative and physiological therapeutics. 1987;10(2):49-55.

146. Mierau D, Cassidy JD, McGregor M, Kirkaldy-Willis WH. A comparison of the effectiveness of spinal manipulative therapy for low back pain patients with and without spondylolisthesis. Journal of Manipulative & Physiological Therapeutics. 1987;10(2):49-55.

147. Miller JS, Litva A, Gabbay M. Motivating patients with shoulder and back pain to self-care: can a videotape of exercise support physiotherapy? Physiotherapy. 2009;95(1):29-35.

148. Minghelli B, Nunes C, Oliveira R. Back School Postural Education Program: Comparison of Two Types of Interventions in Improving Ergonomic Knowledge about Postures and Reducing Low Back Pain in Adolescents. International journal of environmental research and public health. 2021;18(9).

149. Miyamoto GC, Costa LO, Galvanin T, Cabral CM. Efficacy of the addition of modified Pilates exercises to a minimal intervention in patients with chronic low back pain: a randomized controlled trial. Physical therapy. 2013;93(3):310-20.

150. Moffett JK, Jackson DA, Gardiner ED, Torgerson DJ, Coulton S, Eaton S, et al. Randomized trial of two physiotherapy interventions for primary care neck and back pain patients: 'McKenzie' vs brief physiotherapy pain management. Rheumatology. 2006;45(12):1514-21.

151. Molde H, Grasdal A, Eriksen HR. Does early intervention with a light mobilization program reduce long-term sick leave for low back pain: a 3-year follow-up study. Spine. 2003;28(20):2309-15; discussion 16.

152. Moodley M, Brantingham JW. The relative effectiveness of spinal manipulation and ultrasound in mechanical pain: pilot study. Journal of Chiropractic Medicine. 2002;1(4):184-8.

153. Morrison GE, Chase W, Young V, Roberts WL. Back pain: treatment and prevention in a community hospital. Archives of Physical Medicine & Rehabilitation. 1988;69(8):605-9.

154. Moussa WM, Khedr W. Percutaneous radiofrequency facet capsule denervation as an alternative target in lumbar facet syndrome. Clinical Neurology & Neurosurgery. 2016;150:96-104.

155. Murphy SE, Blake C, Power CK, Fullen BM. The effectiveness of a stratified group intervention using the STarTBack screening tool in patients with LBP--a non randomised controlled trial. BMC Musculoskeletal Disorders. 2013;14:342.

156. Murphy SE, Blake C, Power CK, Fullen BM. The effectiveness of a stratified group intervention using the STarTBack screening tool in patients with LBP - A non randomised controlled trial. BMC Musculoskeletal Disorders. 2013;14 (no pagination)(342).

157. Nadler SF, Steiner DJ, Erasala GN, Hengehold DA, Hinkle RT, Beth G, et al. Continuous low-level heat wrap therapy provides more efficacy than Ibuprofen and acetaminophen for acute low back pain. Spine. 2002;27(10):1012-7.

158. Nelson BW, O'Reilly E, Miller M, Hogan M, Wegner JA, Kelly C. The clinical effects of intensive, specific exercise on chronic low back pain: A controlled study of 895 consecutive patients with 1-year follow up. Orthopedics. 1995;18(10):971-81.

159. Nelson CF, Metz RD, LaBrot T. Effects of a managed chiropractic benefit on the use of specific diagnostic and therapeutic procedures in the treatment of low back and neck pain. Journal of Manipulative & Physiological Therapeutics. 2005;28(8):564-9.

160. Nemitalla A, Marco A, Pena C, Leonardo O, De F, Diego G, et al. Kinesio Taping Does Not Provide Additional Benefits in Patients With Chronic Low Back Pain Who Receive Exercise and Manual Therapy: A Randomized Controlled Trial. Journal of Orthopaedic & Sports Physical Therapy. 2016;46(7):506-13.

161. Newman RI, Seres JL, Yospe LP, Garlington B. Multidisciplinary treatment of chronic pain: long-term follow-up of low-back pain patients. Pain. 1978;4(3):283-92.

162. Nordeman L, Nilsson B, Moller M, Gunnarsson R. Early access to physical therapy treatment for subacute low back pain in primary health care: a prospective randomized clinical trial. Clinical Journal of Pain. 2006;22(6):505-11.

163. O'Sullivan PB, Twomey LT, Allison GT. Evaluation of specific stabilizing exercise in the treatment of chronic low back pain with radiologic diagnosis of spondylolysis of spondylolisthesis. Spine. 1997;22(24):2959-67.

164. Overman SS, Larson JW, Dickstein DA, Rockey PH. Physical therapy care for low back pain. Monitored program of first-contact nonphysician care. Physical Therapy. 1988;68(2):199-207.

165. Overmeer T, Boersma K. What Messages Do Patients Remember? Relationships Among Patients' Perceptions of Physical Therapists' Messages, Patient Characteristics, Satisfaction, and Outcome. Physical therapy. 2016;96(3):275-83.

166. Panagopoulos J, Hancock MJ, Ferreira P, Hush J, Petocz P. Does the addition of visceral manipulation alter outcomes for patients with low back pain? A randomized placebo controlled trial. European Journal of Pain. 2015;19(7):899-907.

167. Pereira MG, Roios E, Pereira M. Functional disability in patients with low back pain: the mediator role of suffering and beliefs about pain control in patients receiving physical and chiropractic treatment. Brazilian Journal of Physical Therapy / Revista Brasileira de Fisioterapia. 2017;21(6):465-72.

168. Perich D, Burnett A, O'Sullivan P, Perkin C. Low back pain in adolescent female rowers: A multi-dimensional intervention study. Knee Surgery, Sports Traumatology, Arthroscopy. 2011;19(1):20-9.

169. Perreault K, Dionne CE. Does patient-physiotherapist agreement influence the outcome of low back pain? A prospective cohort study. BMC Musculoskeletal Disorders. 2006;7:76.

170. Pettit J, Glickman-Simon R. Osteopathic Manipulative Therapy for Preterm Infants, Acupuncture for Menopausal Symptoms, Mindfulness-Based Stress Reduction for Chronic Low Back Pain, Chocolate for Ischemic Heart Disease, Berberine for Irritable Bowel Syndrome. Explore. 2016;12(5):388-92.

171. Pivec R, Stokes M, Chitnis AS, Paulino CB, Harwin SF, Mont MA. Clinical and economic impact of TENS in patients with chronic low back pain: analysis of a nationwide database. Orthopedics. 2013;36(12):922-8.

172. Poncela-Skupien C, Pinero-Pinto E, Martinez-Cepa C, Zuil-Escobar Juan C, Romero-Galisteo Rita P, Palomo-Carrion R. How does the Execution of the Pilates Method and Therapeutic Exercise Influence Back Pain and Postural Alignment in Children Who Play String Instruments? A Randomized Controlled Pilot Study. International journal of environmental research and public health. 2020;17(20).

173. Pozo-Cruz BD, Adsuar JC, Parraca J, Pozo-Cruz JD, Moreno A, Gusi N. A web-based intervention to improve and prevent low back pain among office workers: A randomized controlled trial. Journal of Orthopaedic and Sports Physical Therapy. 2012;42(10):831-41.

174. Purepong N, Jitvimonrat A, Boonyong S, Thaveeratitham P, Pensri P. Effect of flexibility exercise on lumbar angle: A study among non-specific low back pain patients. Journal of Bodywork & Movement Therapies. 2012;16(2):236-43.

175. Rajfur J, Pasternok M, Rajfur K, Walewicz K, Fras B, Bolach B, et al. Efficacy of selected electrical therapies on chronic low back pain: A comparative clinical pilot study. Medical Science Monitor. 2017;23:85-100.

176. Rantonen J, Karppinen J, Vehtari A, Luoto S, Viikari-Juntura E, Hupli M, et al. Effectiveness of three interventions for secondary prevention of low back pain in the occupational health setting - a randomised controlled trial with a natural course control. BMC public health. 2018;18(1):598.

177. Ribeiro LH, Jennings F, Jones A, Furtado R, Natour J. Effectiveness of a back school program in low back pain. Clinical & Experimental Rheumatology. 2008;26(1):81-8.

178. Richmond H, Hall AM, Hansen Z, Williamson E, Davies D, Lamb SE. Using mixed methods evaluation to assess the feasibility of online clinical training in evidence based interventions: a case study of cognitive behavioural treatment for low back pain. BMC medical education. 2016;16:163.

179. Rodriguez-Oviedo P, Santiago-Perez MI, Perez-Rios M, Gomez-Fernandez D, Fernandez-Alonso A, Carreira-Nunez I, et al. Backpack weight and back pain reduction: effect of an intervention in adolescents. Pediatric Research. 2018;84(1):34-40.

180. Russell A, Boop FA, Cherny WB, Ligon BL. Neurologic injuries associated with all-terrain vehicles and recommendations for protective measures for the pediatric population. Pediatric Emergency Care. 1998;14(1):31-5.

181. Saner J, Sieben JM, Kool J, Luomajoki H, Bastiaenen CHG, de B, et al. A tailored exercise program versus general exercise for a subgroup of patients with low back pain and movement control impairment: Short-term results of a randomised controlled trial. Journal of Bodywork & Movement Therapies. 2016;20(1):189-202.

182. Schiller L. Effectiveness of spinal manipulative therapy in the treatment of mechanical thoracic spine pain: A pilot randomized clinical trial. Journal of Manipulative and Physiological Therapeutics. 2001;24(6):394-401.

183. Selhorst M, Fischer A, Graft K, Ravindran R, Peters E, Rodenberg R, et al. Timing of Physical Therapy Referral in Adolescent Athletes With Acute Spondylolysis: A Retrospective Chart Review. Clinical Journal of Sport Medicine. 2017;27(3):296-301.

184. Shaw WS, Huang YH. Concerns and expectations about returning to work with low back pain: identifying themes from focus groups and semi-structured interviews. Disability & Rehabilitation. 2005;27(21):1269-81.

185. Shearar KA, Colloca CJ, White HL. A randomized clinical trial of manual versus mechanical force manipulation in the treatment of sacroiliac joint syndrome. Journal of Manipulative & Physiological Therapeutics. 2005;28(7):493-501.

186. Sheets C, Machado LA, Hancock M, Maher C. Can we predict response to the McKenzie method in patients with acute low back pain? A secondary analysis of a randomized controlled trial. European Spine Journal. 2012;21(7):1250-6.

187. Shin JS, Lee J, Lee YJ, Kim MR, Ahn YJ, Park KB, et al. Long-Term Course of Alternative and Integrative Therapy for Lumbar Disc Herniation and Risk Factors for Surgery: A Prospective Observational 5-Year Follow-Up Study. Spine. 2016;41(16):E955-63.

188. Shinozaki T, Yano E, Murata K. Intervention for prevention of low back pain in Japanese forklift workers. American Journal of Industrial Medicine. 2001;40(2):141-4.

189. Slater ME, De L, Campbell K, Lane L, Collins J. Opioids for the management of severe chronic nonmalignant pain in children: A retrospective 1-year practice survey in a children's hospital. Pain Medicine. 2010;11(2):207-14.

190. Smeets RJ, Beelen S, Goossens ME, Schouten EG, Knottnerus JA, Vlaeyen JW. Treatment expectancy and credibility are associated with the outcome of both physical and cognitive-behavioral treatment in chronic low back pain. Clinical Journal of Pain. 2008;24(4):305-15.

191. Smeets RJ, Severens JL, Beelen S, Vlaeyen JW, Knottnerus JA. More is not always better: cost-effectiveness analysis of combined, single behavioral and single physical rehabilitation programs for chronic low back pain. European Journal of Pain. 2009;13(1):71-81.

192. Spinhoven P, Ter K, Kole-Snijders AM, Hutten M, Den O, D J, et al. Catastrophizing and internal pain control as mediators of outcome in the multidisciplinary treatment of chronic low back pain. European Journal of Pain. 2004;8(3):211-9.

193. Staelin R, Koneru SN, Rawe IM. An over-the-counter central sensitization therapy: a chronic back pain registry study of pain relief, medication use and their adverse effects. Pain Management. 2017;7(2):99-111.

194. Stapelfeldt CM, Christiansen DH, Jensen OK, Nielsen CV, Petersen KD, Jensen C. Subgroup analyses on return to work in sick-listed employees with low back pain in a randomised trial comparing brief and multidisciplinary intervention. BMC Musculoskeletal Disorders. 2011;12 (no pagination)(112).

195. Starkweather AR, Coyne P, Lyon DE, Elswick RK, Jr, An K, et al. Decreased low back pain intensity and differential gene expression following Calmare R: results from a double-blinded randomized sham-controlled study. Research in Nursing & Health. 2015;38(1):29-38.

196. Steenstra IA, Anema JR, Bongers PM, de V, H C, Knol DL, et al. The effectiveness of graded activity for low back pain in occupational healthcare. Occupational & Environmental Medicine. 2006;63(11):718-25.

197. Stenner R, Swinkels A, Mitchell T, Palmer S. Exercise prescription for non-specific chronic low back pain (NSCLBP): a qualitative study of patients' experiences of involvement in decision making. Physiotherapy. 2016;102(4):339-44.

198. Sullivan MJ, Stanish WD. Psychologically based occupational rehabilitation: the Pain-Disability Prevention Program. Clinical Journal of Pain. 2003;19(2):97-104.

199. Sutlive TG, Mabry LM, Easterling EJ, Durbin JD, Hanson SL, Wainner RS, et al. Comparison of short-term response to two spinal manipulation techniques for patients with low back pain in a military beneficiary population. Military medicine. 2009;174(7):750-6.

200. Sweetman BJ, Heinrich I, Anderson JAD. A randomized controlled trial of exercises, short wave diathermy, and traction for low back pain, with evidence of diagnosis-related response to treatment. Journal of Orthopaedic Rheumatology. 1993;6(4):159-66.

201. Tella Bosede A, Aiyegbusi Ayoola I, Anikwe Earnest E. EFFICACY OF NERVE FLOSSING TECHNIQUE IN THE MANAGEMENT OF ACUTE SCIATICA. Romanian Journal of Physical Therapy / Revista Romana de Kinetoterapie. 2017;23(39):14-23.

202. Tousignant-Laflamme Y, Bourgault P, Houle S, Lafaille J, Roy J, Roy L. Brief education on chronic low back pain: Brief group education for patients with chronic low back pain - a descriptive study. International Musculoskeletal Medicine. 2013;35(2):65-71.

203. Tsukada M, Takiuchi T, Watanabe K. Low-Intensity Pulsed Ultrasound for Early-Stage Lumbar Spondylolysis in Young Athletes. Clinical Journal of Sport Medicine. 2019;29(4):262-6.

204. Turner JA, LeResche L, Von K, Ehrlich K. Back pain in primary care. Patient characteristics, content of initial visit, and short-term outcomes. Spine. 1998;23(4):463-9.

205. Ugurlu M, Aksekili MAE, Alkan BM, Kara H, Caglar C. Effects of Artcure Diffusional Patch application on pain and functional status in lumbar disc herniation patients: a prospective randomized controlled study. Turkish Journal of Medical Sciences. 2017;47(3):874-82.

206. Ushinohama A, Cunha Bianca P, Costa Leonardo OP, Barela Ana MF, de F, Paulo B. Effect of a single session of ear acupuncture on pain intensity and postural control in individuals with chronic low back pain: a randomized controlled trial. Brazilian Journal of Physical Therapy / Revista Brasileira de Fisioterapia. 2016;20(4):328-35.

207. Valle-Jones JC, Walsh H, O'Hara J, O'Hara H, Davey NB, Hopkin-Richards H. Controlled trial of a back support ('Lumbotrain') in patients with non-specific low back pain. Current Medical Research and Opinion. 1992;12(9):604-13.

208. van der R, van T, Barendse J, Knol D, van M, de V. Intensive group training protocol versus guideline physiotherapy for patients with chronic low back pain: a randomised controlled trial. European Spine Journal. 2008;17(9):1193-200.

209. van der R, van T, van M, de V. Economic evaluation of an intensive group training protocol compared with usual care physiotherapy in patients with chronic low back pain. Spine. 2008;33(4):445-51.

210. Vas J, Aguilar I, Perea-Milla E, Mendez C. Effectiveness of acupuncture and related techniques in treating non-oncological pain in primary healthcare - An audit. [Spanish, English] Eficacia de la acupuntura y sus tecnicas relacionadas para el tratamiento del dolor no oncologico en atencion primaria: una auditoria medica. Revista Internacional de Acupuntura. 2008;2(1):56-62.

211. Veselsky J, Hudeckova L, Mayzlik J. [Experiences with lumbosacral manipulation therapy in an industrial polyclinic]. Acta Chirurgiae Orthopaedicae et Traumatologiae Cechoslovaca. 1968;35(5):421-4.

212. Vidal J, Borras PA, Ponseti FJ, Cantallops J, Ortega FB, Palou P. Effects of a postural education program on school backpack habits related to low back pain in children. European Spine Journal. 2013;22(4):782-7.

213. Voerman JS, Remerie S, Westendorp T, Timman R, Busschbach JJV, Passchier J, et al. Effects of a Guided Internet-Delivered Self-Help Intervention for Adolescents with Chronic Pain. Journal of Pain. 2015;16(11):1115-26.

214. Vong SK, Cheing GL, Chan F, So EM, Chan CC. Motivational enhancement therapy in addition to physical therapy improves motivational factors and treatment outcomes in people with low back pain: a randomized controlled trial. Archives of Physical Medicine & Rehabilitation. 2011;92(2):176-83.

215. Wand BM, Abbaszadeh S, Smith AJ, Catley MJ, Moseley GL. Acupuncture applied as a sensory discrimination training tool decreases movement-related pain in patients with chronic low back pain more than acupuncture alone: a randomised cross-over experiment. British Journal of Sports Medicine. 2013;47(17):1085-9.

216. Wang LQ. [Observation on therapeutic effects of scraping therapy and warming acupuncture-moxibustion on 50 cases of fasciitis of back muscles]. Zhongguo Zhenjiu. 2006;26(7):478-80.

217. Wang PC, Ritz BR, Janowitz I, Harrison RJ, Yu F, Chan J, et al. A randomized controlled trial of chair interventions on back and hip pain among sewing machine operators: the los angeles garment study. Journal of Occupational & Environmental Medicine. 2008;50(3):255-62.

218. Wang QG, Lin XY, Yan XX, Liu NG. [Comparative observation of efficacy on lumbar disc herniation treated with acupotomology and operation]. Zhongguo Zhenjiu. 2011;31(8):743-6.

219. Wang YL. [Observation on the therapeutic effect of lumbar disc herniation treated with different acupuncture therapies]. [Chinese]. Zhongguo zhen jiu = Chinese acupuncture & moxibustion. 2013;33(7):605-8.

220. Ward J, Tyer K, Coats J, Purmoghaddam A, Amonette W. CASE SERIES OF SYMPTOMATOLOGY COMPRESSION RATES OF CHIROPRACTIC PATIENTS WITH ACUTE LOW BACK PAIN AT 2-WEEKS AND 4-WEEKS. Chiropractic Journal of Australia. 2017;45(4):289-303.

221. Waterworth RF, Hunter IA. An open study of diflunisal, conservative and manipulative therapy in the management of acute mechanical low back pain. New Zealand Medical Journal. 1985;98(779):372-5.

222. Wen YL, He C, Huang M, Liang XS. [Observation on therapeutic effect of the third lumbar transverse process syndrome treated with acupotomy and blood pricking therapy]. Zhongguo Zhenjiu. 2012;32(4):345-8.

223. Werneke Mark W, Edmond S, Deutscher D, Ward J, Grigsby D, Young M, et al. Effect of Adding McKenzie Syndrome, Centralization, Directional Preference, and Psychosocial Classification Variables to a Risk-Adjusted Model Predicting Functional Status Outcomes for Patients With Lumbar Impairments. Journal of Orthopaedic & Sports Physical Therapy. 2016;46(9):726-41.

224. West DT, Mathews RS, Miller MR, Kent GM. Effective management of spinal pain in one hundred seventy-seven patients evaluated for manipulation under anesthesia. Journal of Manipulative & Physiological Therapeutics. 1999;22(5):299-308.

225. Wilkinson MJ. Does 48 hours' bed rest influence the outcome of acute low back pain? British Journal of General Practice. 1995;45(398):481-4.

226. Williams NH, Edwards RT, Linck P, Muntz R, Hibbs R, Wilkinson C, et al. Cost-utility analysis of osteopathy in primary care: Results from a pragmatic randomized controlled trial. Family Practice. 2004;21(6):643-50.

227. Williams NH, Wilkinson C, Russell I, Edwards RT, Hibbs R, Linck P, et al. Randomized osteopathic manipulation study (ROMANS): Pragmatic trial for spinal pain in primary care. Family Practice. 2003;20(6):662-9.

228. Wilson FA, Licciardone JC, Kearns CM, Akuoko M. Analysis of provider specialties in the treatment of patients with clinically diagnosed back and joint problems. Journal of Evaluation in Clinical Practice. 2015;21(5):952-7.

229. Winter S. Effectiveness of targeted home-based hip exercises in individuals with non-specific chronic or recurrent low back pain with reduced hip mobility: A randomised trial. Journal of back and musculoskeletal rehabilitation. 2015;28(4):811-25.

230. Xinliang L, Hanney William J, Masaracchio M, Kolber Morey J, Mei Z, Spaulding Aaron C, et al. Immediate Physical Therapy Initiation in Patients With Acute Low Back Pain Is Associated With a Reduction in Downstream Health Care Utilization and Costs. Physical Therapy. 2018;98(5):336-47.

231. Yang LY, Lu DJ, Li YH. [Observation on therapeutic effect of fire-needle therapy on lumbar intervertebral disc herniation]. Zhongguo Zhenjiu. 2009;29(6):449-51.

232. Yu PF, Jiang FD, Liu JT, Jiang H. Outcomes of conservative treatment for ruptured lumbar disc herniation. Acta Orthopaedica Belgica. 2013;79(6):726-30.

233. Zapata KA, Wang-Price SS, Fletcher TS, Johnston CE. Factors influencing adherence to an app-based exercise program in adolescents with painful hyperkyphosis. Scoliosis and Spinal Disorders. 2018;13 (1) (no pagination)(11).

234. Zhao F, Cao DB, Yuan YQ, Luo J, Wen YY, Wang Y, et al. [Efficacy observation of nonspecific low back pain treated with the dragon-tiger fighting needling method]. Zhongguo Zhenjiu. 2012;32(6):507-10.

235. Zhao Y, Wang GL. [Randomized controlled study on proximal needling for sciatica]. Zhongguo Zhenjiu. 2011;31(5):425-8.

***Ineligible intervention (2 full text articles)***

1. Fritz JM, Clifford SN. Low back pain in adolescents: a comparison of clinical outcomes in sports participants and nonparticipants. Journal of athletic training. 2010;45(1):61-6.

2. Seitsalo S. Operative and conservative treatment of moderate spondylolisthesis in young patients. The Journal of bone and joint surgery British volume. 1990;72(5):908-13.

***Ineligible outcome (3 full text articles)***

1. Czaprowski D, Leszczewska J, Kolwicz A, Pawlowska P, Afeltowicz-Mich A, Sitarski D, et al. Comparison of influence of three physiotherapeutic techniques: postisometric relaxation, kinetic control and stabilization exercises on the flexibility of pelvic girdle muscles: prospective, randomized, single blinded study. Studies in Health Technology & Informatics. 2012;176:499-.

2. Fairweather MM, Sidaway B. Ideokinetic imagery as a postural development technique. Research quarterly for exercise and sport. 1993;64(4):385-92.

3. Tsukada M, Takiuchi T, Watanabe K. Low-Intensity Pulsed Ultrasound for Early-Stage Lumbar Spondylolysis in Young Athletes. Clinical journal of sport medicine : official journal of the Canadian Academy of Sport Medicine. 2019;29(4):262-6.

***Ineligible study design (35 full text articles)***

1. Clinical presentation and treatment outcomes of children and adolescents with low back pain in physical therapy. Dissertation Abstracts International: Section B: The Sciences and Engineering. 2010;70(10-B).

2. Actrn. Chiropractic for Adolescent Low back pain including Manipulation/Manual Therapy. The CALM Study. Chiropractic for Adolescent Low back pain including Manipulation/Manual Therapy compared to a sham treatment for pain and disability A randomised controlled trial pilot study The CALM Study. 2015.

3. Alcantara J, Ohm J, Kunz D. Treatment-related aggravations, complications and improvements attributed to chiropractic spinal manipulative therapy of paediatric patients: a survey of parents...14th Annual Symposium on Complementary Health Care, 11th to 13th December 2007, University of Exeter, UK. Focus on Alternative & Complementary Therapies. 2007;12:4-.

4. Anonymous. Summaries for patients. Identifying patients with low back pain who are likely to benefit from spinal manipulation. Annals of internal medicine. 2004;141(12):I39.

5. Beneux J, Rigault P, Pouliquen JC, Padovani JP, Guyonvarch G. [Lumbo-sacral spondylolysis and spondylolisthesis in children. Study of 82 cases]. Annales de Pediatrie. 1976;23(2):135-41.

6. Beneux J, Rigault P, Pouliquen JC, Padovani JP, Guyonvarch G. [Lumbo-sacral spondylolysis and spondylolisthesis in children. Study of 82 cases]. Spondylolyse et spondylolisthesis lombo-sacre chez l'enfant Etude de 82 observations. 1976;23(2):135-41.

7. Bobaly V, Brigitta S, Gabriella K, Eleonora L, Pongrac A, Andras O, et al. Application and examination of the efficiency of a core stability training program among dancers. European Journal of Integrative Medicine. 2016;11.

8. Broadhurst NA. Pelvic dysfunction. Journal of Neurological and Orthopaedic Medicine and Surgery. 1994;15(3):127-9.

9. Bronfort G, Evans RHMLBSC. Spinal manipulation and exercise for adolescent low back pain. 2017;17.

10. Bush T, Cherkin D, Barlow W. The impact of physician attitudes on patient satisfaction with care for low back pain. Archives of family medicine. 1993;2(3):301-5.

11. Cannon SR, James SE. Back pain in athletes. British Journal of Sports Medicine. 1984;18(3):159-64.

12. Chen S. The clustered needling, massage and cupping used for treatment of obstinate myofascitis of the back--a report of 68 cases. Journal of Traditional Chinese Medicine. 2007;27(2):113-4.

13. Ciccarelli M, Fraser K, Vaz S. Allied health management of technology-related musculoskeletal complaints among children and adolescents. Australian occupational therapy journal. 2016;63(6):399-407.

14. Cichoke AJ. For the kids' sake. Chiropr. 1989;2(2):55.

15. Czarnecki ML, Garwood MM, Weisman SJ. Advanced practice nurse-directed telephone management of acute pain following pediatric spinal fusion surgery. Journal for Specialists in Pediatric Nursing. 2007;12(3):159-69.

16. Diener HC. [Backache: early physical therapy has little effect]. MMW Fortschritte der Medizin. 2016;158(5):44.

17. Dissing KB, Vach W, Hartvigsen J, Wedderkopp N, Hestbæk L. Potential treatment effect modifiers for manipulative therapy for children complaining of spinal pain. Secondary analyses of a randomised controlled trial. Chiropr & Manual Ther. 2019;27(59):Online access only 11 p.

18. Ernst E, Kissling R. A positive trial of homoeopathy for low back pain. Focus on Alternative & Complementary Therapies. 2003;8(2):205-6.

19. Evans R, Haas MLBHLSCBG. Spinal manipulation and exercise for low back pain in adolescents: a randomized trial. 2018;7:200.

20. Hayden JA, Mior SA, Verhoef MJ. Evaluation of chiropractic management of pediatric patients with low back pain: A prospective cohort study. Journal of Manipulative and Physiological Therapeutics. 2003;26(1):1-8.

21. Hermus J, Hulsbosch M, Guldemond N, v R. Effectiveness and quality of life of a new TLSO compared with the Boston brace. Studies in Health Technology & Informatics. 2010;158:315-.

22. Horowitz S. Evidence-based indications for therapeutic massage. Alternative & Complementary Therapies. 2007;13(1):30-5.

23. Jorda L, Perez B, Garcia-Mifsud M, Jimeno B, Ortiz H, Castells A. [Back school: a simple way to improve pain and postural behaviour]. Anales de Pediatria. 2014;81(2):92-8.

24. Lavigne V. Weight limit recommendation in backpack use for school-aged children. J Clin Chiropr Pediatr. 2014;14(2):1156-9.

25. Leonidou A, Lepetsos P, Pagkalos J, Antonis K, Flieger I, Tsiridis E, et al. Treatment for spondylolysis and spondylolisthesis in children. Journal of orthopaedic surgery (Hong Kong). 2015;23(3):379-82.

26. Long CR. Effectiveness of spinal manipulative therapy in the treatment of mechanical thoracic spine pain: a pilot randomized clinical trial. Journal of Manipulative & Physiological Therapeutics. 2002;25(8):538.

27. Nashat SM, Abdelwahab MM, Almowalad AM, Yousef AH, Alkanali AA, Sharaf DK, et al. Attitude, believes and decision of practitioners associated with complementary and alternative therapy in the treatment of pediatric population. International Research Journal of Pharmacy. 2018;9(7):126-30.

28. Oishi A, Sodeyama T, Yanagisawa S. An investigation of manual therapy treatment for juvenile spondylolysis. Physiotherapy (United Kingdom). 2015;101(SUPPL. 1):eS1127.

29. Puglisi JA, Powers AK, Monroe R, Leonard JR. Sacroiliac joint pain in pediatric and adolescent patients...2010 Combined Sections Meeting (CSM), San Diego, California, February 17-20, 2010. Journal of Orthopaedic & Sports Physical Therapy. 2010;40(1):A21-A.

30. Russell A, Boop FA, Cherny WB, Ligon BL. Neurologic injuries associated with all-terrain vehicles and recommendations for protective measures for the pediatric population. Pediatric Emergency Care. 1998;14(1):31-5.

31. Sawni A, Thomas R. Pediatricians' attitudes, experience and referral patterns regarding complementary/alternative medicine: A national survey. BMC Complementary and Alternative Medicine. 2007;7 (no pagination)(18).

32. Selhorst M, Martin L, Ravindran R, MacDonald J, Rodenberg R, Krishnamurthy R, et al. A Pilot Study Assessing An Immediate Functional Progression Program In Adolescent Athletes With A Spondylolysis. Medicine & Science in Sports & Exercise. 2021;53(8S):375-.

33. Sousa A, Mateus ARSACJAPMPAM. Benefits of the clinical pilates program in lower back pain in young handball players. 2019;51:S219.

34. Standaert CJ. Bed rest or continuation of activity for acute low back pain? Clinical Journal of Sport Medicine. 2003;13(4):275.

35. Strikovic V, Krasnik R, Zvekic-Svorcan J, Demesi D, Kuhajda D, Ivanic J, et al. Potential risk factors for back pain in children. Journal of Back and Musculoskeletal Rehabilitation. 2019;32(5):749-54.

***Duplicates (7 full text articles)***

1. Ahlqwist A, SÄLlfors C. Experiences of low back pain in adolescents in relation to physiotherapy intervention. International Journal of Qualitative Studies on Health & Well-Being. 2012;7:1-11.

2. Evans R, Haas MSCLBHLBG. Spinal manipulation and exercise for low back pain in adolescents: a randomized trial. 2018;159:1297.

3. Gonzalez-Galvez N, Marcos-Pardo Pablo J, Albaladejo-Saura M, Lopez-Vivancos A, Vaquero-Cristobal R. Effects of a Pilates programme in spinal curvatures and hamstring extensibility in adolescents with thoracic hyperkyphosis: a randomised controlled trial. Postgraduate medical journal. 2023;99(1171):433-41.

4. Jones MA, Stratton G, Reilly T, Unnithan VB. Recurrent non-specific low-back pain in adolescents: The role of exercise. Ergonomics. 2007;50(10):1680-8.

5. Ng L, Caneiro JP, Campbell A, Smith A, Burnett A, O'Sullivan P. Cognitive functional approach to manage low back pain in male adolescent rowers: a randomised controlled trial. British journal of sports medicine. 2015;49(17):1125-31.

6. Selhorst M, Selhorst B. Lumbar manipulation and exercise for the treatment of acute low back pain in adolescents: a randomized controlled trial. 2015;23:226.

7. Selhorst M, Selhorst B. Lumbar manipulation and exercise for the treatment of acute low back pain in adolescents: A randomized controlled trial. Journal of Manual and Manipulative Therapy. 2015;23(4):226-33.

***Full text excluded from checking reference lists (5 full text articles)***

*Ineligible population (3 full text articles)*

1. Saarni LA, Rimpelä AH, Nummi TH, Kaukiainen A, Salminen JJ, Nygård C-H. 2009. Do ergonomically designed school workstations decrease musculoskeletal symptoms in children? A 26-month prospective follow-up study. Applied Ergonomics 40(3) 491–499 10.1016/j.apergo.2008.09.011

2. Geldhof, Elisabeth, et al. "Effects of a two-school-year multifactorial back education program in elementary schoolchildren." *Spine* 31.17 (2006): 1965-1973.

3. Batistão MV, Carnaz L, Moreira R de FC, Sato T de O, Carnaz L, Moreira R de FC, et al. Effects of a muscular stretching and strengthening school‐ based exercise program on posture, trunk mobility, and musculoskeletal pain among elementary schoolchildren ‐ a randomized controlled trial. Fisioter Em Mov. 2019;32:1–9.

*Ineligible study design (1 full text article)*

1. Mueller, Juliane, et al. "Effects of six-month trunk stability exercises on low back pain prevalence in young athletes." *MEDICINE AND SCIENCE IN SPORTS AND EXERCISE*. Vol. 44. 530 WALNUT ST, PHILADELPHIA, PA 19106-3621 USA: LIPPINCOTT WILLIAMS & WILKINS, 2012.

*Cannot retrieve (1 full text article)*

1. Goodgold, Shelley A. "Backpack intelligence: Implementation of a backpack safety program with fifth grade students." *Orthop Pract* 15 (2003): 15-20.
