# Supplementary material for: Rehabilitation of back pain in the pediatric population: a mixed studies systematic review
Source: Chiropr Man Therap. 2024 May 8;32:14. doi: 10.1186/s12998-024-00538-z (PMC11080233; doi:10.1186/s12998-024-00538-z)
Supplement: Supplementary file 4 — Additional file 4: Evidence Profile. [file 12998_2024_538_MOESM4_ESM.docx]

**Additional file 4. Evidence Profile of Included Studies**

**Author(s):**

**Question:** Individualized physical therapy + exercise compared to No treatment/no additional treatment for children with back pain (Ahlqwist)

**Setting:**

**Bibliography:**

| **Certainty assessment** | | | | | | | **№ of patients** | | **Effect** | | **Certainty** | **Importance** |
| --- | --- | --- | --- | --- | --- | --- | --- | --- | --- | --- | --- | --- |
| **№ of studies** | **Study design** | **Risk of bias** | **Inconsistency** | **Indirectness** | **Imprecision** | **Other considerations** | **Individualized physical therapy + exercise** | **No treatment/no additional treatment** | **Relative (95% CI)** | **Absolute (95% CI)** |  |  |
| **Pain intensity (follow-up: immediately post 12 week intervention; assessed with: VAS; benefit indicated by lower values; Scale from: 0 to 10)** | | | | | | | | | | | | |
| 1^1^ | randomised trials | very serious^a^ | not serious^b^ | not serious^c^ | very serious^d^ | none | 23 | 22 | - | MD **0.5 lower** (3.9 lower to 2.9 higher) | ⨁◯◯◯ Very low | CRITICAL |
| **Disability (follow-up: immediately post 12 week intervention; assessed with: RMDQ; benefit indicated by lower values; Scale from: 0 to 24)** | | | | | | | | | | | | |
| 1^1^ | randomised trials | very serious^a^ | not serious^b^ | not serious^c^ | not serious^e^ | none | 23 | 22 | - | MD **0.8 lower** (2.31 lower to 0.7 higher) | ⨁⨁◯◯ Low | CRITICAL |
| **Quality of life - physical functioning (follow-up: immediately post 12 week intervention; assessed with: CHQ-CF87; benefit indicated by higher values)** | | | | | | | | | | | | |
| 1^1^ | randomised trials | very serious^a^ | not serious^b^ | not serious^c^ | very serious^f^ | none | Post-intervention difference in mean change: 3.4 (p>0.05) (no statistically significant difference) | | | | ⨁◯◯◯ Very low | CRITICAL |
| **Quality of life - role emotional (follow-up: immediately post 12 week intervention; assessed with: CHQ-CF87; benefit indicated by higher values)** | | | | | | | | | | | | |
| 1^1^ | randomised trials | very serious^a^ | not serious^b^ | not serious^c^ | very serious^f^ | none | Post-intervention difference in mean change: 1.2 (p>0.05) (no statistically significant difference) | | | | ⨁◯◯◯ Very low | CRITICAL |
| **Quality of life - role behavioural (follow-up: immediately post 12 week intervention; assessed with: CHQ-CF87; benefit indicated by higher values)** | | | | | | | | | | | | |
| 1^1^ | randomised trials | very serious^a^ | not serious^b^ | not serious^c^ | very serious^f^ | none | Post-intervention difference in mean change: -0.7 (p>0.05) (no statistically significant difference) | | | | ⨁◯◯◯ Very low | CRITICAL |
| **Quality of life - role physical (follow-up: immediately post 12 week intervention; assessed with: CHQ-CF87; benefit indicated by higher values)** | | | | | | | | | | | | |
| 1^1^ | randomised trials | very serious^a^ | not serious^b^ | not serious^c^ | very serious^f^ | none | Post-intervention difference in mean change: 5.4 (p>0.05) (no statistically significant difference) | | | | ⨁◯◯◯ Very low | CRITICAL |
| **Quality of life - role emotional (follow-up: immediately post 12 week intervention; assessed with: CHQ-CF87; benefit indicated by higher values)** | | | | | | | | | | | | |
| 1^1^ | randomised trials | very serious^a^ | not serious^b^ | not serious^c^ | very serious^f^ | none | Post-intervention difference in mean change: 1.2 (p>0.05) (no statistically significant difference) | | | | ⨁◯◯◯ Very low | CRITICAL |
| **Quality of life - bodily pain (follow-up: immediately post 12 week intervention; assessed with: CHQ-CF87; benefit indicated by higher values)** | | | | | | | | | | | | |
| 1^1^ | randomised trials | very serious^a^ | not serious^b^ | not serious^c^ | very serious^f^ | none | Post-intervention difference in mean change: 8.2 (p>0.05) (no statistically significant difference) | | | | ⨁◯◯◯ Very low | CRITICAL |
| **Quality of life - behaviour (follow-up: immediately post 12 week intervention; assessed with: CHQ-CF87; benefit indicated by higher values)** | | | | | | | | | | | | |
| 1^1^ | randomised trials | very serious^a^ | not serious^b^ | not serious^c^ | very serious^f^ | none | Post-intervention difference in mean change: -1.2 (p>0.05) (no statistically significant difference) | | | | ⨁◯◯◯ Very low | CRITICAL |
| **Quality of life - mental health (follow-up: immediately post 12 week intervention; assessed with: CHQ-CF87; benefit indicated by higher values)** | | | | | | | | | | | | |
| 1^1^ | randomised trials | very serious^a^ | not serious^b^ | not serious^c^ | very serious^f^ | none | Post-intervention difference in mean change: 2.5 (p>0.05) (favouring individualized physical therapy) | | | | ⨁◯◯◯ Very low | CRITICAL |
| **Quality of life - general health (follow-up: immediately post 12 week intervention; assessed with: CHQ-CF87; benefit indicated by higher values)** | | | | | | | | | | | | |
| 1^1^ | randomised trials | very serious^a^ | not serious^b^ | not serious^c^ | very serious^f^ | none | Post-intervention difference in mean change: 1.2 (p>0.05) (no statistically significant difference) | | | | ⨁◯◯◯ Very low | CRITICAL |
| **Quality of life - change in health (follow-up: immediately post 12 week intervention; assessed with: CHQ-CF87; benefit indicated by higher values)** | | | | | | | | | | | | |
| 1^1^ | randomised trials | very serious^a^ | not serious^b^ | not serious^c^ | very serious^f^ | none | Post-intervention difference in mean change: 2.1 (p>0.05) (no statistically significant difference) | | | | ⨁◯◯◯ Very low | CRITICAL |
| **Quality of life - family activities (follow-up: immediately post 12 week intervention; assessed with: CHQ-CF87; benefit indicated by higher values)** | | | | | | | | | | | | |
| 1^1^ | randomised trials | very serious^a^ | not serious^b^ | not serious^c^ | very serious^f^ | none | Post-intervention difference in mean change: -4.6 (p>0.05) (no statistically significant difference) | | | | ⨁◯◯◯ Very low | CRITICAL |
| **Quality of life - family cohesion (follow-up: immediately post 12 week intervention; assessed with: CHQ-CF87; benefit indicated by higher values)** | | | | | | | | | | | | |
| 1^1^ | randomised trials | very serious^a^ | not serious^b^ | not serious^c^ | very serious^f^ | none | Post-intervention difference in mean change: -0.6 (p>0.05) (no statistically significant difference) | | | | ⨁◯◯◯ Very low | CRITICAL |

**CHQ-CF87:** Child Health Questionnaire-Child Form 87**; CI:** confidence interval; **MD:** mean difference; **RMDQ:** Roland Morris Disability Questionnaire; **VAS:** visual analog scale

#### Explanations

a. Risk of bias assessed using the Cochrane risk-of-bias tool for randomized trials (ROB1). Options are not serious, serious (rate quality of evidence down one level), and very serious (rate quality of evidence down two levels). Not serious: study rated as ‘low risk of bias’ or ‘unclear’ (that are not accounted for below). Serious: study with concerns regarding unbalanced baseline characteristics between groups, unclear co-interventions, high/unbalanced drop-out and/or unclear intention-to-treat analysis. (blinding rated as Unclear). Very serious: study with concerns regarding unclear randomization sequence generation, inadequate allocation concealment and/or lack of blinding (blinding rated as high ROB).

b. Inconsistency assessed using effect size variance in direction or magnitude. Options are not serious, serious (rate quality of evidence down one level), and very serious (rate quality of evidence down two levels). Single study, does not apply.

c. Indirectness results if the patients, interventions, or outcomes are different from the research question under investigation. Options are not serious, serious (rate quality of evidence down one level), and very serious (rate quality of evidence down two levels).

d. Imprecision assessed using between-group effect [point estimate (95% CI)]. Options are not serious, serious (rate quality of evidence down one level), and very serious (rate quality of evidence down two levels). For ‘very serious’: similar to ‘serious’ but to a greater extent. If the point estimate is or is not clinically significant: the CI crosses the boundaries of both appreciable harm and benefit (i.e., very wide CI).

e. Imprecision assessed using between-group effect [point estimate (95% CI)]. Options are not serious, serious (rate quality of evidence down one level), and very serious (rate quality of evidence down two levels). For ‘not serious’: If the point estimate is not clinically significant: the upper and lower boundaries of the CI do not cross a clinically significant threshold; the CI may cross the null as long as neither boundary crosses a clinically significant threshold. If the point estimate is clinically significant: the CI does not cross the null and the boundaries do not cross a clinically significant threshold.

f. Imprecision cannot be determined due to lack of information (SD/CIs) provided by study authors.

#### References

1.Ahlqwist A, Hagman M,Kjellby-Wendt G,Beckung E. Physical Therapy Treatment of Back Complaints on Children and Adolescents. Spine; 2008.
